# Learning to suppress a distractor may not be unconscious

**DOI:** 10.3758/s13414-022-02608-x

**Published:** 2022-11-23

**Authors:** Francisco Vicente-Conesa, Tamara Giménez-Fernández, David Luque, Miguel A. Vadillo

**Affiliations:** 1 Departamento de Psicología Básica, Facultad de Psicología, Universidad Autónoma de Madrid, 28049 Madrid, Spain; 2 Departamento de Psicología Básica, Universidad de Málaga, Malaga, Spain

**Keywords:** Additional singleton task, Awareness, Distractor suppression, Implicit learning

## Abstract

The additional singleton task has become a popular paradigm to explore visual statistical learning and selective attention. In this task, participants are instructed to find a different-shaped target among a series of distractors as fast as possible. In some trials, the search display includes a singleton distractor with a different color, making search more difficult. This singleton distractor appears more often in one location than in the remaining locations. The typical results of these experiments show that participants learn to ignore the area of the screen that is more likely to contain the singleton distractor. It is often claimed that this learning takes place unconsciously, because at the end of the experiment participants seem to be unable to identify the location where the singleton distractor appeared most frequently during the task. In the present study, we tested participants’ awareness in three high-powered experiments using alternative measures. Contrary to previous studies, the results show clear evidence of explicit knowledge about which area of the display was more likely to contain the singleton distractor, suggesting that this type of learning might not be unconscious.

**Supplementary Information:**

The online version contains supplementary material available at 10.3758/s13414-022-02608-x.

## Introduction

Visual attention is extremely sensitive to previous experience. Over the last 20 years, researchers have developed numerous experimental paradigms to explore how statistical regularities in the environment shape different aspects of visual attention like, for instance, our ability to find particular objects in a scene or to ignore irrelevant stimuli (Chun & Jiang, 1998; Ferrante et al., 2018; Geng & Behrmann, 2002; Goschy et al., 2014; Jiang, 2018; Sauter et al., 2018; Wang & Theeuwes, 2018a, 2018b). The consensus arising from this body of research is that visual statistical learning is a fast and efficient process that can take place unconsciously, in the sense that participants cannot verbalize the regularities that drive their performance in these tasks and cannot use this knowledge flexibly in other tasks (Goujon et al., 2015; Jiang & Sisk, 2019; Theeuwes, 2018).

Consider, for instance, a popular paradigm known as *contextual cuing* (Chun & Jiang, 1998). In a typical experiment, participants are instructed to find a tilted T among several L-shaped distractors. Some of the displays are presented repeatedly across blocks, whereas the rest of the displays are generated randomly on each block and only appear once during the experiment. Participants usually show faster search times for repeated displays compared to random displays. However, this effect seems to take place outside participants’ awareness. For instance, participants’ ability to discriminate between repeated and new displays is rarely above chance (e.g., Annac et al., 2013; Chun & Jiang, 2003; Manginelli et al., 2013) and, in addition, their performance in this recognition task is usually uncorrelated with the search advantage for repeated displays (Colagiuri & Livesey, 2016). These results are taken as evidence that contextual cuing takes place unconsciously or at least depends on mechanisms that do not rely on explicit knowledge (Goujon et al., 2015; Jiang & Sisk, 2019).

Statistical learning can also bias attention toward a particular spatial region of the search display that has contained the target frequently in the past. For example, in a popular paradigm known as *probabilistic cuing* or *location probability learning* (Geng & Behrmann, 2002; Jiang, Swallow et al., 2013a; Jiang et al., 2014), the target appears more often in a specific quadrant of the screen (i.e., the rich quadrant) than in the others (i.e., the sparse quadrants). The usual finding is that participants need less time to find the target when it appears in the rich quadrant. As in the case of contextual cuing, this effect has been understood as the result of implicit or unconscious learning (e.g., Jiang, Swallow et al., 2013a). In these studies, participants are typically asked to report if they believe that the target appeared more often in one quadrant than in the others and then they are asked to select the quadrant that, in their opinion, contained the target most frequently. Their responses to both questions usually reveal no evidence of explicit learning (e.g., Jiang, Capistrano, et al., 2013a; Jiang, Sha, et al., 2018).

**Fig. 1:**
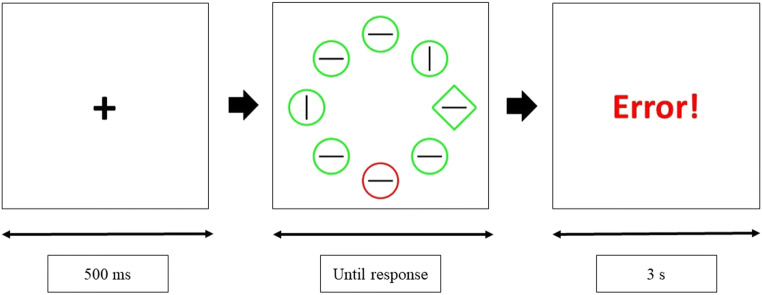
Sequence of events in each visual search trial of Experiments 1–3. In this particular example, the green diamond is the target and the red circle is the singleton distractor. Participants are instructed to find and respond to the orientation of the black line inside the target. A corrective feedback appears during 3 s in case of error

Visual statistical learning does not only facilitate target selection; it can also improve distractor suppression (Goschy et al., 2014; Ferrante et al., 2018; Sauter et al., 2018). The *additional singleton task* has become one of the most popular experimental paradigms to explore this type of attentional bias (e.g., Wang & Theeuwes, 2018a, 2018b). In this task, participants are instructed to find a singleton target among several different-shaped distractors (e.g., a green diamond among green circles, see Fig. 1). In some trials, a highly salient singleton distractor is added to the search display (e.g., a red circle among green stimuli). Unknown to participants, the singleton distractor appears more frequently in one particular location (i.e., the high-probability distractor location or HPDL) than in the others (i.e., the low-probability distractor locations or LPDL). Many studies show that, eventually, participants learn to suppress attention to the location where the singleton distractor is most likely to appear (e.g., Gao & Theeuwes, 2020; Wang & Theeuwes, 2018a, 2018b). For example, participants’ responses are faster when the singleton distractor appears in the HPDL. In contrast, they are slower when the target appears in the HPDL. Moreover, there is a spatial gradient in the magnitude of this reaction-time (RT) benefit around the HPDL, that is, RTs decrease gradually as the target appears in locations increasingly distant from the HPDL.

As in the case of contextual cuing and probabilistic cuing, distractor suppression in the additional singleton task is thought to be driven by unconscious learning. In most of these studies, awareness tests performed after the search task usually suggest that participants are not aware of the statistical regularities that drive performance in the task, even if they benefit from them (Gao & Theeuwes, 2020; Wang & Theeuwes, 2018a, 2018b). In the typical awareness tests employed in this task (see Table S1 in the Online Supplementary Material (OSM) for a comprehensive list of the methods used in the literature), participants are first asked if they have noticed any statistical regularity during the task. For example, they may be asked if they think that the singleton distractor appeared more frequently in a particular location. Immediately afterwards, and regardless of their response to the first question, they are asked to select the location where they think the distractor appeared most frequently. Participants’ responses to these questions usually reveal little knowledge of the task structure and, furthermore, removing the few subjects who select the correct location or show high confidence in their choice does not seem to affect the results of response time analyses. Based on this evidence, it is often concluded that this kind of learning must be unconscious.

Converging evidence for the unconscious nature of distractor suppression comes from studies showing that performance in the additional singleton task is insensitive to explicit instructions provided to the participants (Wang & Theeuwes, 2018a). Also, distractor suppression seems to be unaffected by manipulations of the availability of cognitive resources (Gao & Theeuwes, 2020). However, only a handful of experiments have tested these claims and, in the vast majority of studies, the conclusion that distractor suppression is unconscious is based solely on the fact that participants perform poorly in the awareness test and that removing those participants who could have some awareness does not affect the results. We return to this issue in the *General discussion*.

The conclusion that contextual cuing, probabilistic cuing and distractor suppression in the additional singleton task are driven by unconscious learning has important implications for current theories of attentional control. Traditionally, attentional control was thought to depend on either stimulus-driven (i.e., the salience of stimuli) or goal-directed processes^1^ (i.e., participants’ conscious goals; Vecera et al., 2014). The guidance of attention in visual statistical learning paradigms like the ones discussed above cannot be based on stimulus-driven control, because these effects do not depend on the salience of stimuli. At the same time, it has been argued that they cannot depend on goal-directed processes, because if participants lack any reportable knowledge of the regularities that drive performance, these effects must be independent of a conscious intention to pay attention to some objects but not others. Following this reasoning, recent theories of attention posit that attentional control may sometimes depend on a third type of process, usually referred to as selection history (Awh et al., 2012; Theeuwes, 2018). From this point of view, people do not only pay attention to salient or task-relevant stimuli, but also to stimuli that have been attended in the past, even if they are no longer relevant for the current task and if devoting attention to them actually hinders performance. As contextual cuing, probabilistic cuing and distractor inhibition in the additional singleton task have been considered to take place outside participants’ awareness, these effects are ideal examples of selection history and, not surprisingly, they are cited recurrently in this literature (Theeuwes, 2018, 2019).

The conclusion that visual statistical learning is independent from participants’ awareness might be premature, though. Recent research reveals important methodological shortcomings in the typical awareness tests included in some of these tasks and in the statistical analyses conducted on participants’ responses, questioning the conclusion that these effects are unconscious. For example, Vadillo et al. (2020) found significant levels of awareness in the probabilistic cuing task at the meta-analytical level, although most individual studies were underpowered to detect it. Furthermore, experiments employing alternative, and arguably more sensitive, methods to measure participants’ awareness usually find clear evidence of explicit knowledge (e.g., Giménez-Fernández et al., 2020; Vadillo et al., 2020). Meta-analyses and empirical studies with the contextual cuing paradigm arrive at the same conclusion: Participants’ performance in the awareness test is systematically above chance when the measure of awareness is sufficiently sensitive and the study is properly powered to detect such effects (Geyer et al., 2020; Smyth & Shanks, 2008; Vadillo et al., 2016, 2022).

As in the case of contextual cuing and probabilistic cuing, in our opinion there are good reasons to suspect that the measures and analyses used in the additional singleton task might be underestimating participants’ awareness. The awareness tests usually employed in this task are similar to those used in probabilistic cuing and, therefore, they are likely to suffer from the same methodological shortcomings. The main goal of the present study is to explore this possibility using more sensitive measures of awareness, larger samples, and fully preregistered experimental protocols.

## Overview of the present experiments

In the following experiments, we tested three different measures of awareness. In all cases, participants performed an additional singleton task in which the location of the singleton distractor was manipulated and then completed an awareness test with two questions. The first question, inspired by Wang and Theeuwes (2018a), asked participants to report if they had noticed any statistical regularity regarding the location of the singleton distractor. Although responses to this question are usually provided by means of a yes/no choice, recent experiments measuring awareness in the probabilistic cuing task (Giménez-Fernández et al., 2020) suggest that a rating scale may be more sensitive than a dichotomous question. Consequently, in the present experiments, participants responded to this test using a rating scale.

The second question of the awareness test was different in each of the current experiments. In most previous studies (Gao & Theeuwes, 2020; Wang & Theeuwes, 2018a, 2018b), participants were asked to choose the location that they believed had contained the singleton distractor most frequently. Experiment 1 in the present article also included this test. In Experiment 2, we asked participants to rank the three locations that they believed had contained the singleton distractor most often during the visual search task. Previous experiments with the probabilistic cuing task show that this type of ranking test might provide a more sensitive measure of awareness than the traditional test used in Experiment 1 (Giménez-Fernández et al., 2020; Vadillo et al., 2020). Despite this, the ranking test might still miss an important piece of information about participants’ knowledge: Even if two participants provide identical responses in the ranking test, they can still differ in the extent to which they believe that the distribution of distractors was biased towards a particular location. For instance, one of them might think that the HPDL contained the singleton distractor in 50% of the trials while the other one believes that this happened in 30% of the trials. To overcome this problem, in Experiment 3 participants had to estimate how many times they believed that the singleton distractor had appeared in each location of the display. The methods and analysis plan of Experiments 1–3 were preregistered before data collection at https://osf.io/9vfrc, https://osf.io/qhwrm, and https://osf.io/dx6ez, respectively.

## Experiment 1

As mentioned above, the conclusion that distractor suppression depends on unconscious processes is often based on two arguments: (1) that performance in the awareness test is poor, and (2) that removing participants who show some evidence of awareness (i.e., participants who select the HPDL in the awareness test) makes no difference in the results observed in the behavioral task (Ferrante et al., 2018; Gao & Theeuwes, 2020; Geng & Berhmann, 2002; Jiang, Li et al., 2015; Jiang, Swallow, & Rosenbaum, 2013b; Jiang, Swallow, Rosenbaum et al., 2013c; Wang & Theeuwes, 2018a, 2018b). From our point of view, both strategies are problematic. The fact that, as a group, participants fail to perform above chance in the awareness test might be entirely due to insufficient statistical power (Dienes, 2015; Vadillo et al., 2016, 2020). Selecting participants who seem to perform poorly in the awareness test is also a cause for concern. This strategy assumes that performance in the awareness test is a perfect indicator (i.e., reliable and valid) of participants’ conscious knowledge. But the evidence collected in similar implicit learning paradigms shows that the reliability of awareness test is usually quite low (Malejka et al., 2021; Vadillo et al., 2020, 2022) and that, consequently, many participants classified as “unaware” are likely to have some explicit knowledge (Rothkirch et al., 2022; Shanks, 2017).

Experiment 1 was conceived as a conceptual replication of the typical studies conducted with this paradigm (e.g., Gao & Theeuwes, 2020; Wang & Theeuwes, 2018a, 2018b) specifically aimed at addressing the two problems highlighted above. In contrast with the majority of previous studies, we recruited a relatively large sample to ensure that the absence of evidence of consciousness in these studies is not due to a simple lack of statistical power. This was not only intended to improve the sensitivity of the experiment to detect above-chance performance in the awareness test, but also to achieve sufficient power to test the hypothesis that even participants who fail to identify the HPDL in the awareness test still may have some level of awareness. In particular, we predict that these participants will be more likely to select a location that is close to the HPDL than more distant locations.

### Method

#### Participants and apparatus

Although Experiment 1 is similar to previous experiments with this task, some of the analyses that we planned to conduct were new in this literature (e.g., the analysis of the pattern of responses in the awareness test within participants who failed to select the HPDL). Therefore, we could not plan our sample size based on an effect-size estimate from previous research. In the absence of better evidence, we found it appropriate to set the sample size to 80 participants per experiment. This sample is considerably larger than the typical samples used in previous experiments with the additional singleton task (23 participants on average in Gao & Theeuwes, 2020; Wang & Theeuwes, 2018a; 2018b) and grants 90% power to detect a small-to-medium effect of *d*_z_ = 0.37 in a two-tailed paired-samples *t*-test.

Participants in Experiments 1–3 were psychology students at the Autonomous University of Madrid (UAM), and they participated in exchange for course credits. They performed the experimental task in groups of four participants in a laboratory with individual cubicles. All participants provided informed consent. The UAM ethics committee approved the experimental protocols of Experiments 1–3 (Ref. CEI-80-1473). All the experiments were carried out following the sanitary protocols recommended by the UAM.

#### Procedure and design

Unless noted otherwise, the procedure and design of the Experiment 1 were the same as in Wang and Theeuwes (2018b) and very similar to other studies using the additional singleton task (Lin et al., 2021). Each trial started with a 500-ms fixation cross in the center of the screen. Then, a search display with eight figures forming an imaginary circle around the center of the screen was presented until participant’s response (see Fig. 1). In all trials, the target was a stimulus with a unique shape. That is, the search display could consist of a diamond (target) among circles (distractors) or vice versa. A target was present in all trials. In some trials, all the figures were presented in the same color (green or red). In other trials, there was a distractor with a unique color (i.e., the singleton distractor), either red among green figures or vice versa. Inside each figure, there was a line segment, vertical or horizontal. Participants were asked to find the target as fast as possible and respond with the left arrow key if the line segment inside the target was horizontal, or with the up arrow key if the line segment was vertical. A corrective feedback was presented on the screen for 3 s if they made an error. The intertrial interval was 500 ms.

Each block included 120 trials. In 40 trials (33%) all the figures were presented in the same color (no-distractor condition or ND); in 52 (43%) trials a distractor was presented on a particular location (high-probability condition or HPDL); and in the remaining 28 trials (24%) the distractor was presented equally often at the other seven locations (low-probability condition or LPDL). Note that, given these probabilities, the singleton distractor appeared on the HPDL in 65% of distractor-present trials. Trials were presented in random order within each block. The target appeared in a random location not occupied by the singleton distractor. The HPDL was randomly chosen for each participant and remained the same for each participant during the task.

Participants completed six blocks and had the opportunity to take a short break after each block. During the resting breaks, a message on the screen indicated that they could rest for any amount of time they wanted and that they could resume the task when they were ready by pressing the space key. The first 20 trials of the experiment were not included in the analyses. Before the first block, participants completed three practice trials to get used to the task displays.

Crucially, after finishing the visual search task, participants completed an awareness test similar to the one conducted in previous studies (Wang & Theeuwes, 2018a, 2018b). The awareness test included two questions. Firstly, participants were asked if they had noticed any regularity in the location of the distractor. The specific text of this question was “You may have noticed that, in many trials, a figure appeared in a different color than the rest. Do you think that this figure tended to appear more often in a particular location of the screen?” As noted in the introduction, in previous studies, participants are typically asked to answer this question with “yes” or “no” (Gao & Theeuwes, 2020; Wang & Theeuwes, 2018a, 2018b). However, evidence from previous studies (Giménez-Fernández et al., 2020) suggests that a rating scale may be more sensitive. Hence, participants were asked to respond using a scale ranging from 1 to 6, labelled as “definitely not,” “probably not,” “possibly not,” “possibly yes,” “probably yes,” and “definitely yes.” Secondly, right after this, and regardless of their response, they were asked to choose the location of the search display where they believed the distractor had appeared most often. To do this, we showed them eight placeholders (green circles) forming an imaginary circle around the center of the screen, exactly as in a standard trial. Every circle was labelled with a number, from 1 to 8, and participants entered their response by writing the number corresponding to the location where they thought the distractor had appeared most often. Then, the circle corresponding with the chosen location changed its color, so that the participants could identify their choice clearly, and they were given the chance to change their response, if they wanted to do so. In the actual experiment, all the materials used in the awareness test were presented in Spanish. The original texts used in this and the following experiments are reproduced verbatim in the OSM.

### Results and discussion

#### Data pre-processing

Unless noted otherwise, all the analyses reported below (including data pre-processing) follow the preregistered protocol. One participant failed to complete the experiment due to a technical problem. The preregistered protocol established that only participants with an overall accuracy above 75% of in the search task would be included in the analyses, but in fact, all participants achieved accuracies above this selection criterion. For the analysis of RT, only correctly responded trials were included in the analyses. RTs lower than 200 ms or higher than 5 s were also removed. Then, for each participant, we calculated the mean and standard deviation of RTs in the remaining trials and excluded trials 2.5 standard deviations above and below the mean.

#### Attentional capture

**Fig. 2:**
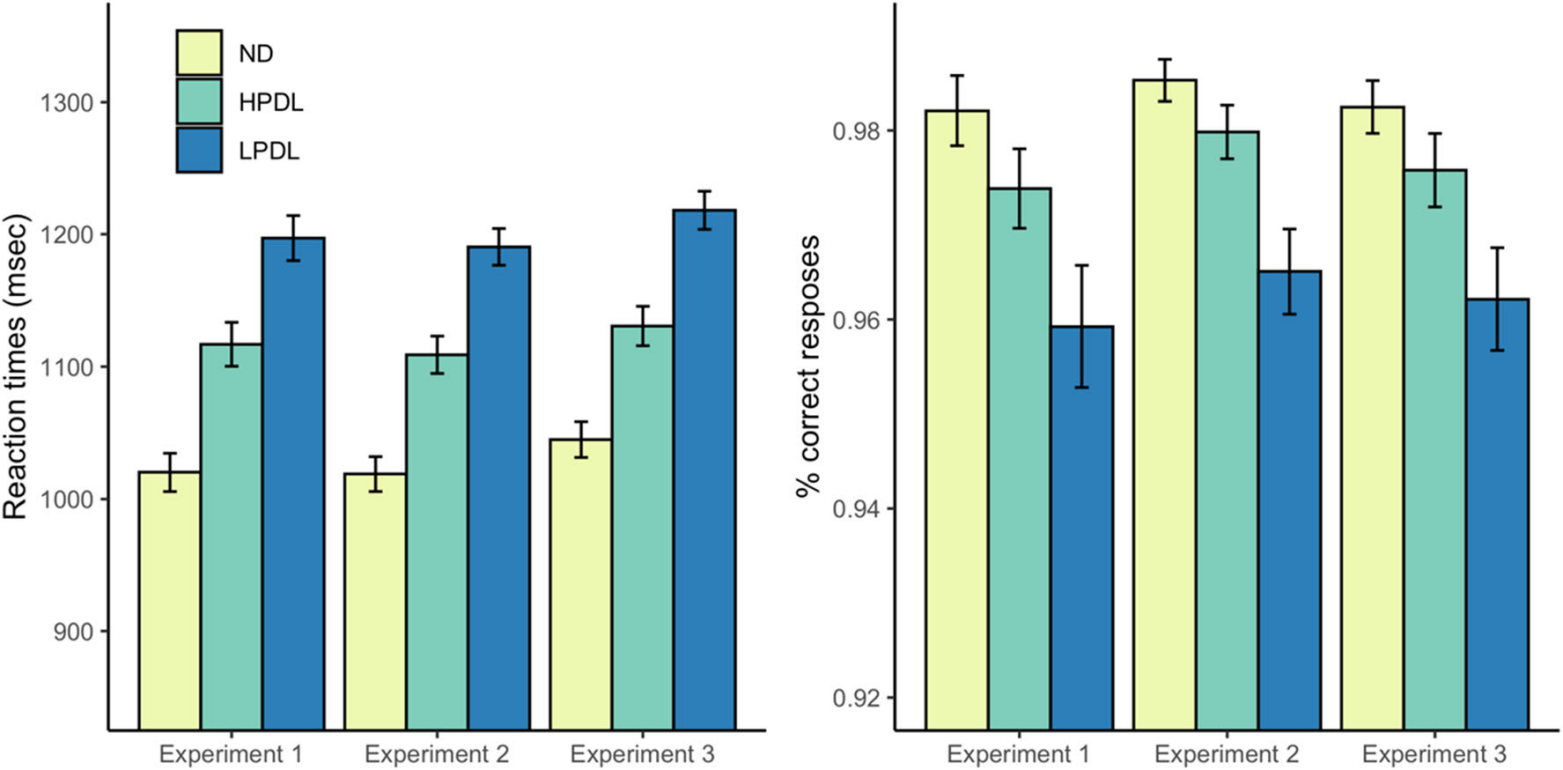
Mean reaction times (**left panel**) and proportion of correct responses (**right panel**) in trials without the singleton distractor (ND), in trials with the singleton distractor located in the high-probability distractor location (HPDL), and with the singleton distractor located in any of the remaining locations (low probability distractor location or LPDL), separately for Experiments 1–3. Error bars in this and all subsequent figures denote correlation- and difference-adjusted 95% confidence intervals for within-participants designs, following Cousineau et al. (2021)

Figure 2 shows the mean RTs and proportion of correct responses in the three experimental conditions. A one-way repeated-measures ANOVA on mean RTs with the experimental condition as the only factor (no-distractor vs. singleton distractor in the high-probability location vs. singleton distractor in the low-probability location) yielded a significant result, *F*(2, 156) = 275.37, *p* < .001, ${\upeta}_{\mathrm{p}}^2$ = .78. RTs were 96.89 ms faster in the no-distractor condition than in the HPDL condition, *t*(78) = 12.92, *p* < .001, *d*_z_ = 1.45, and these, in turn, were 80.13 ms faster than in the LPDL condition, *t*(78) = 14.58, *p* < .001, *d*_z_ = 1.64. RTs were also significantly faster in the no-distractor than in the LPDL condition, *t*(78) = 19.22, *p* < .001, *d*_z_ = 2.16. The same analyses were also conducted on the proportion of correct responses (see Fig. 2, right panel). The one-way ANOVA revealed a significant effect of condition, *F*(2, 156) = 46.31, *p* < .001, ${\upeta}_{\mathrm{p}}^2$ = .37. The proportion of correct responses was significantly higher in the no-distractor than in the HPDL condition, *t*(78) = 5.13, *p* < .001, *d*_z_ = 0.57, and significantly higher in the HPDL than in the LPDL condition, *t*(78) = 5.93, *p* < .001, *d*_z_ = 0.66. The difference between the no-distractor and the LPDL conditions was also significant, *t*(78) = 7.75, *p* < .001, *d*_z_ = 0.87. Overall, these results confirm that participants were slower and made more errors when the search display included a singleton distractor, but this effect was partially ameliorated when the singleton distractor appeared in the high-probability location.

#### Target suppression at the high-probability distractor location (HPDL)

Although these analyses were not preregistered, we also tested whether participants’ ability to find the target in no-distractor trials deteriorated when the target was presented in the HPDL. This analysis confirmed that RTs were 59 ms slower when the target was presented in the HPDL, compared to the remaining locations, *t*(78) = 4.79, *p* < .001, *d*_z_ = .54, although response accuracy was similar in both types of trials, *t*(78) = 0.27, *p* = .792, *d*_z_ = .03. These results are consistent with the conclusion that participants were actively suppressing attention to the HPDL and had more difficulties to find the target when presented at that location (for a similar result, see Allenmark et al., 2019; Wang & Theewes, 2018a, 2018b; Zhang et al., 2019).

#### Spatial gradient of the suppression effect

**Fig. 3:**
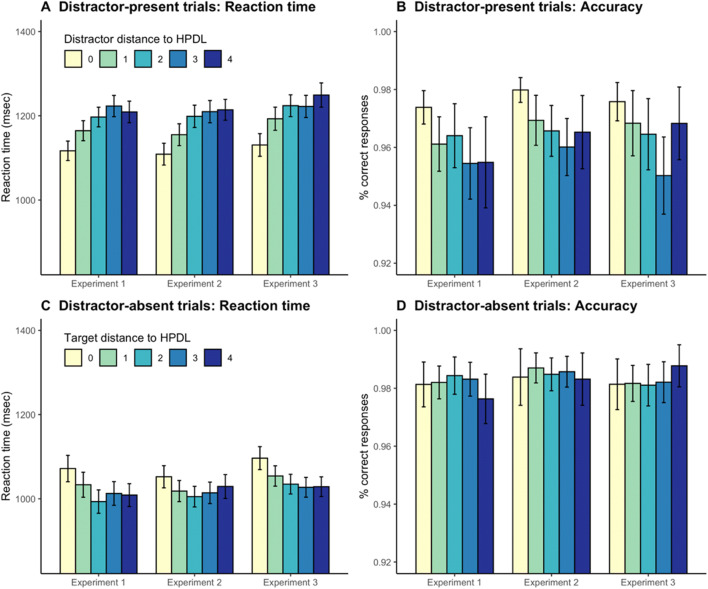
Reaction times and accuracy rates in distractor-present trials (panels **A** and **B**) as a function of the distance between singleton distractor location and the high-probability distractor location (HPDL), and in distractor-absent trials (panels **C** and **D**) as a function of the distance between the target and the HPDL

The next analysis compared RTs and error rates in trials where the singleton distractor appeared at different locations to explore the spatial gradient in the distribution of attention. Following Wang and Theeuwes (2018b), we grouped trials in five categories (0–4) according to the distance of the singleton distractor from the HPDL. In these analyses, 0 corresponds to the HPDL, 1 corresponds to the two locations immediately next to the HPDL, and so on. Mean RTs and accuracy rates as a function of distractor distance are shown in Fig. 3 (panels A and B). We carried out two one-way ANOVAs on RTs and response accuracy with location as a factor. These ANOVAs revealed a significant effect of distance on RTs, *F*(4, 312) = 32.21, *p* < .001, ${\upeta}_{\mathrm{p}}^2$ = .29, and also on the proportion of correct responses, *F*(4, 312) = 4.51, *p* = .001, ${\upeta}_{\mathrm{p}}^2$ = .05. We also explored the effect of distance on RTs with a linear function, contrasting the slope against zero to explore the spatial gradient of search times.^2^ We fitted linear mixed-effects models predicting RTs (or accuracies) from the distance of the singleton distractor to the HPDL, adding a random intercept for participants. These analyses confirmed that RTs increased with distance, *b* = 24.31, *t*(315) = 9.97, *p* < .001, while accuracy rates decreased significantly, *b* = -0.004, *t*(315) = 3.77, *p* < .001. Overall, these results suggest that the suppression of the singleton distractor became progressively easier as the distractor location approached the HPDL.

We also explored if the location of the target made a difference in distractor-absent trials. Mean RTs and accuracy rates as a function of target distance are shown in Fig. 3 (panels C and D). Two one-way ANOVAs with the distance between the target location and the HPDL confirmed that distance had a significant impact on RTs, *F*(4, 312) = 13.63, *p* < .001, ${\upeta}_{\mathrm{p}}^2$ = .15, but the effect failed to reach significance for accuracy rates, *F*(4, 312) = 1.90, *p* = .109, ${\upeta}_{\mathrm{p}}^2$ = .023. This null result is probably due to the fact that participants’ performance was almost at ceiling in distractor-absent trials. Linear mixed-effects models with participant-wise random intercepts confirmed that RTs decreased as the target was presented further away from the HPDL, *b* = -14.67, *t*(315) = 5.49, *p* < .001, but target location made no significant difference in accuracy, *b* = -0.0008, *t*(315) = -1.25, *p* = .211. In general, these results confirm that when the search displays did not include a singleton distractor, participants needed more time to find the target if it appeared in the location usually occupied by the salient distractor or close to it, presumably because participants were actively ignoring these locations.

Following Wang and Theeuwes (2018b), the preregistered protocol of Experiments 1–3 also included an exploration of feature-based inter-trial priming effects. For the sake of brevity, these analyses are only reported in the OSM. To summarize the main outcome of these analyses, we found that feature-based priming played only a small role in visual search, which reached statistical significance in Experiments 2 and 3, but not in Experiment 1. Overall, these results support the conclusion that participants’ performance in the task was mainly driven by the location of the stimuli and not by other features, such as their color or shape.

#### Awareness test

**Fig. 4:**
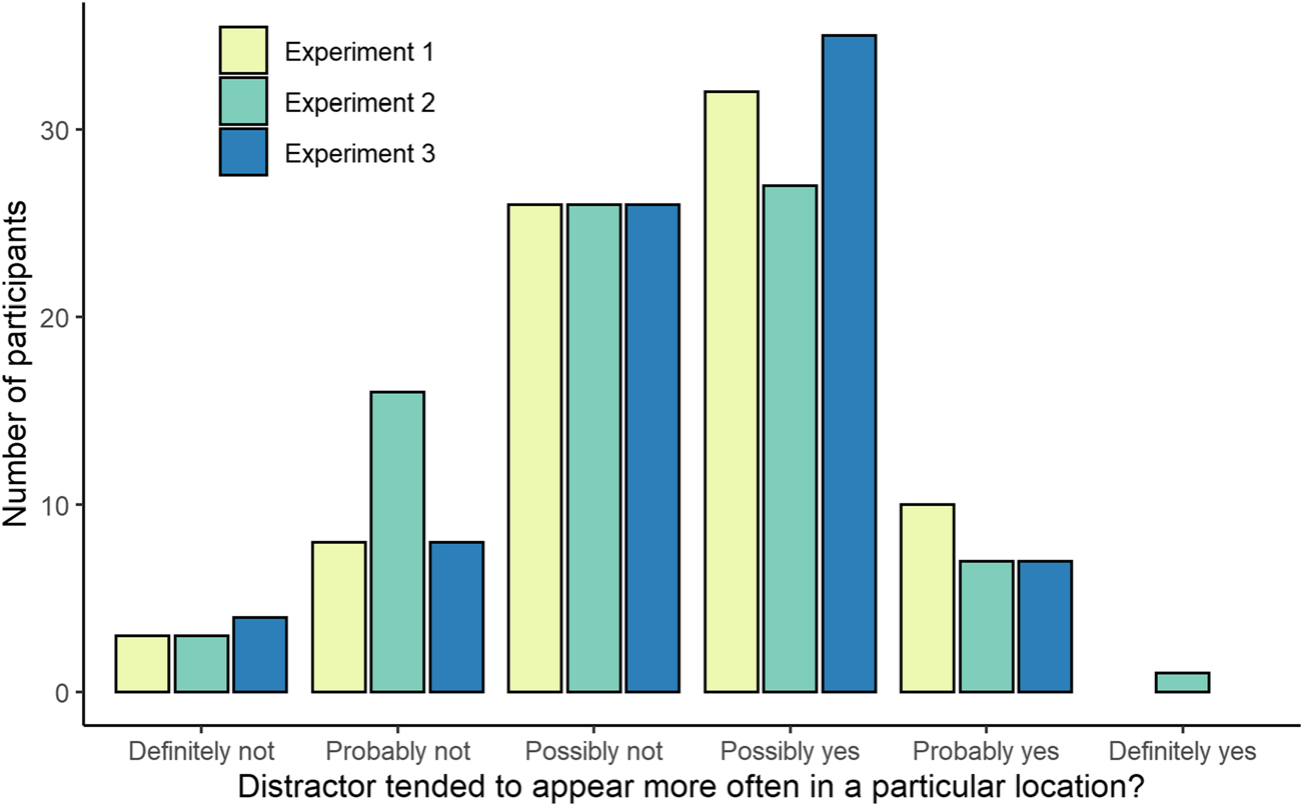
Participants’ responses to the first question of the awareness test

Figure 4 summarizes the responses provided by participants in response to the first question of the awareness test. When asked whether they thought that the singleton distractor had appeared more frequently in one particular location, the modal response was “Possibly yes” (4 in a scale from 1 to 6). If these responses are interpreted as numerical values, the average response was 3.48, 95% CI [3.26, 3.70]. In general, participants showed little confidence in their responses, in either direction.

**Fig. 5:**
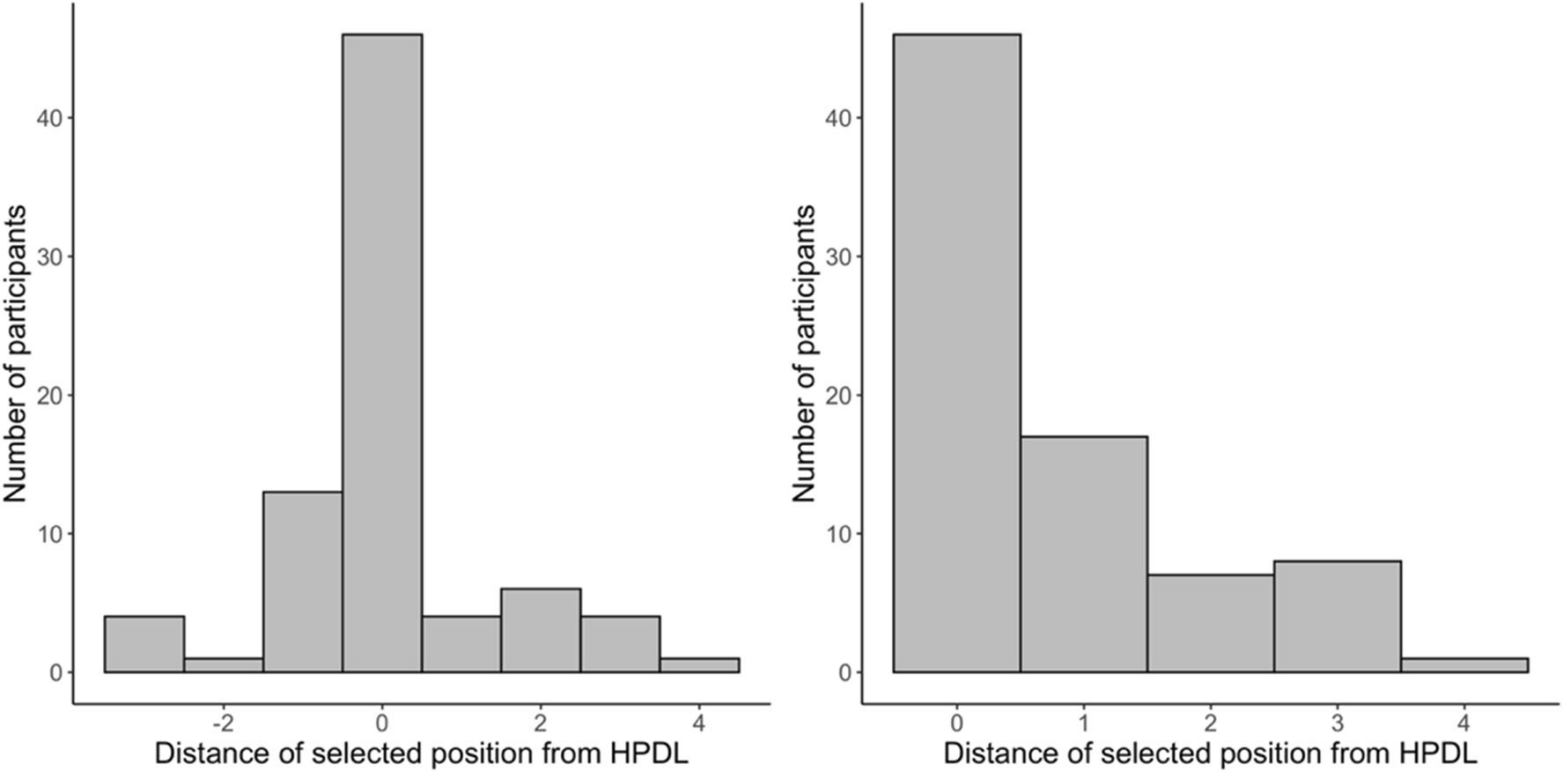
Participants’ responses to the second question of the awareness test in Experiment 1. The left panel shows the distribution of participants’ choices, where 0 represents the high-probability distractor location (HPDL), and values 1–4 represent progressively distant locations. Positive and negative values indicate clockwise or counter-clockwise positions. The right panel shows participants’ choices, regardless of whether they were on the clockwise or the counter-clockwise side of the HPDL

The second question of the awareness test asked participants to select the location that, in their opinion, had contained the singleton distractor most often. Our hypothesis was that these responses would not be randomly distributed, even within the subgroup of participants that gave an incorrect response. The left panel of Fig. 5 shows the distribution of participants’ choices, where 0 represents the high-probability distractor location, and values 1–4 represent progressively distant locations. The sign (negative or positive) denotes whether the location was on the clockwise side (positive) of the HLDP or on the counter-clockwise side (negative). As can be seen, a majority of participants selected the HPDL. Following the preregistered plan, we conducted a chi-squared analysis with the participants’ choices as an eight-level factor to check if the distribution of the answers departed from chance. The results confirmed that participants choices were not random, *χ*^2^(7) = 161.1, *p* < .001. A binomial test confirmed that the proportion of participants who provided the correct response, 58%, was also significantly higher than chance, *p* < .001.

Finally, we also tested whether there was a spatial gradient in the responses provided by participants who failed to select the HPDL. If these participants were truly unaware of the regularity in the distractor location, then all responses should be equally likely. However, we anticipated that even participants who select the wrong location would be more likely to select a location close to the HPDL than a far location. This would suggest that even participants who failed the traditional awareness test probably had some knowledge of the HPDL. The right panel of Fig. 5 shows participants’ choices, this time ignoring whether they were on the clockwise or the counter-clockwise side of the HPDL. As predicted, even among participants who failed to identify the HPDL, locations close to the HPDL were more likely to be selected than locations further away from the HPDL. In the preregistered protocol, we planned to run a chi-squared analysis with the distance of the chosen location from the HPDL location (1 vs. 2 vs. 3) as a factor, after excluding participants who selected the HPDL location and also those who selected the location at distance 4. We intended to ignore the latter because there are two possible locations at distances 1, 2, and 3 (i.e., clockwise and counter-clockwise from the HPDL) but there is only one location at distance 4 (i.e., the location opposite to the HPDL). Therefore, the number of distance-4 choices is not directly comparable to distances 1–3. This test approached but failed to reach statistical significance, *χ*^2^(2) = 5.69, *p* = .058. Following the suggestion of a reviewer, to include distance 4 in the analysis, we run a non-registered analysis comparing the proportion of participants selecting distances 1–4, but assuming that, by chance, participants would be twice as likely to select distances 1, 2, or 3 than distance 4. This test did yield significant results, *χ*^2^(3) = 9.85, *p* = .002.

Overall, these analyses show that, although participants showed little confidence in their judgments about whether the singleton distractor tended to appear in a specific location or not, they were well above chance when asked to identify the HPDL. More than half of them provided the correct answer. Furthermore, those who failed to identify the HPDL did not perform randomly in the awareness test. Most of them selected the location immediately next to the HPDL and only one out of 79 participants selected the location opposite to the HPDL.

## Experiment 2

Experiment 2 was identical to Experiment 1 except for the second question of the awareness test. Studies conducted in our lab with the probabilistic cuing task (Giménez-Fernández et al., 2020; Vadillo et al., 2020) suggest that asking participants to rank the locations depending on the frequency with which they contained the singleton distractor may be a more sensitive measure than asking them to select just one location. The logic for using this alternative measure is that even if participants fail to select the HPDL as their first choice, they might still be likely to rank it higher than other locations. The present study tested the role of awareness in the additional singleton task with this alternative method.

### Method

#### Participants, apparatus, procedure, and design

As mentioned above all the details of the procedure, design, and sample size planning were identical to Experiment 1. Therefore, 80 participants were tested in this study. All the selection criteria and resources used to carry out the study were the same as in Experiment 1. The first stage of the study (i.e., the visual search task), was identical to Experiment 1. The first question of the awareness test was also exactly as in Experiment 1. For the second question, participants were asked to select the three locations that, in their opinion, had contained the singleton distractor most frequently during the visual search task. This test comprised three separate trials or questions. In the first one, they were asked: “At this point, if you had to choose a location as the one that contained the figure of a different color most frequently, which one would it be?” Right after this, they were asked: "Ignoring your previous choice, what is the next location that in your opinion contained most frequently the figure of a different color?” Before concluding, they were asked: "Finally, ignoring your previous choices, what is the next location that in your opinion contained the figure of a different color most frequently?" This allowed us to examine if participants had an approximate idea of which region of the screen was more likely to contain the singleton distractor, beyond merely selecting a specific location. At the end of the test, participants were given the opportunity to change their answers, if they wanted to do so.

### Results and discussion

#### Attentional capture

The same participant and trial selection performed in Experiment 1 were applied to the visual search task in Experiment 2. None of the 80 participants failed to meet the selection criterion. The average RTs and accuracy rates in the three experimental conditions (no-distractor vs. singleton distractor in the high-probability location vs. singleton distractor in the low-probability location) are shown in Fig. 2. One-way ANOVAs confirmed that, as in Experiment 1, there were significant differences among the three conditions in RTs, *F*(2, 158) = 393.65, *p* < .001, ${\upeta}_{\mathrm{p}}^2$ = .83, and accuracy rates, *F*(2, 158) = 68.11, *p* < .001, ${\upeta}_{\mathrm{p}}^2$ = .46. Participants were 90 ms faster in the no-distractor condition than in the HPDL condition, *t*(79) = 15.95, *p* < .001, *d*_z_ = 1.78. They were also significantly more accurate, *t*(79) = 4.49, *p* < .001, *d*_z_ = 0.50. Participants were also 81 ms faster in the HPDL condition than in the LPDL condition, *t*(79) = 14.77, *p* < .001, *d*_z_ = 1.65, and they were also more accurate, *t*(79) = 7.23, *p* < .001, *d*_z_ = 0.80. Participants were also significantly faster, *t*(79) = 24.28, *p* < .001, *d*_z_ = 2.71, and more accurate, *t*(79) = 10.12, *p* < .001, *d*_z_ = 1.13, in the no-distractor than in the LPDL condition. These results replicate those of Experiment 1.

#### Target suppression at the HPDL

As in Experiment 1, we ran two non-registered analyses to test whether performance in no-distractor trials deteriorated when the target was presented in the HPDL. RTs were 38 ms slower when the target was presented in the HPDL, compared to the remaining locations, *t*(79) = 4.33, *p* < .001, *d*_z_ = 0.48, although response accuracy was comparable in both types of trials, *t*(79) = 0.45, *p* = .656, *d*_z_ = 0.05.

#### Spatial gradient of the suppression effect

As in Experiment 1, we tested whether there was a spatial gradient in the suppression of the singleton distractor. Figure 3 (panels A and B) shows RTs and accuracy rates in distractor-present trials as a function of the distance between the location of the distractor and the HPDL. Two one-way ANOVAs confirmed that, as in Experiment 1, RTs were significantly different depending on the location of the singleton distractor, *F*(4, 316) = 40.54, *p* < .001, ${\upeta}_{\mathrm{p}}^2$ = .34, and the same applied to accuracy rates, *F*(4, 316) = 5.80, *p* < .001, ${\upeta}_{\mathrm{p}}^2$ = .07. Linear mixed-effects models with random intercepts for participants, confirmed that RTs increased as the distractor location departed from the HPDL, *b* = 26.56, *t*(319) = 11.60, *p* < .001 and accuracy rates, in contrast, decreased, *b* = -0.003, *t*(319) = 3.94, *p* < .001.

We also tested whether there was a spatial gradient in RTs and accuracy rates in distractor-absent trials depending on the location of the target (panels C and D in Fig. 2). The location of the target made a significant difference in RTs, *F*(4, 316) = 5.28, *p* < .001, but not in accuracy, *F*(4, 316) = 0.43, *p* = .785, ${\upeta}_{\mathrm{p}}^2$ < .01. Note that, as in Experiment 1, participants’ accuracy was at ceiling in these trials, regardless of target location, and therefore this null result is difficult to interpret. Linear mixed-effects models with random intercepts for participants detected a decrease in RTs as the target location deviated from the HPDL, *b* = -5.03, *t*(319) = 1.97, *p* = .049, but no such effect was found for accuracy rates, *b* = -0.0003, *t*(319) = -0.37, *p* = .711. These results replicate those of Experiment 1.

#### Awareness test

The responses provided by participants in response to the first question of the awareness test are shown in Fig. 4. As in Experiment 1, when asked whether they thought that the singleton tended to appear in a particular location, the modal response was “Possibly yes” (4, on a scale from 1 to 6). The average response was 3.28, 95% CI [3.04, 3.51].

**Fig. 6:**
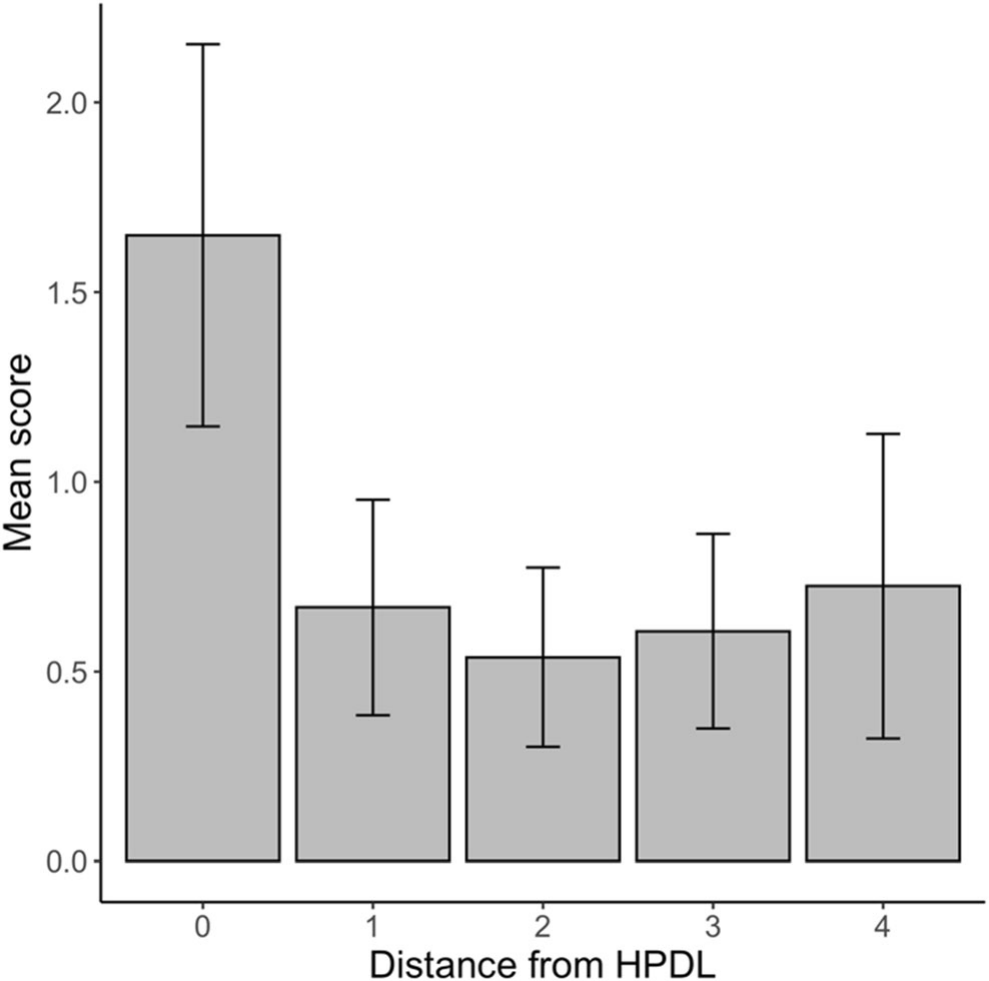
Distribution of participants’ responses to the second question of the awareness test in Experiment 2. Note that for distances 1–3 each participant contributed with two different scores. To build the present figure, before averaging scores across participants, we first averaged scores for the two locations with the same distance from the high-probability distractor location (HPDL) within each participant

Following the preregistered protocol, we assigned a score to each location depending on participants’ responses. The first location chosen by participants received a score of 3; the second and third ones were scored as 2 and 1, respectively; and all other locations received a score of 0. We predicted that there would be a relationship between the score obtained by a location and its distance from the HPDL. The distribution of scores obtained by each location is shown in Fig. 6. Consistent with our hypothesis, a linear mixed-effects model with random intercepts for participants found that the score received by each location decreased as the distance from the HPDL increased, *b* = -0.165, *t*(638) = 4.75, *p* < .001. These results confirm that, on average, participants knew which location was more likely to contain the singleton distractor.

## Experiment 3

In Experiment 2, participants were asked to rank the three locations that, in their opinion, had contained the singleton distractor most frequently during the visual search task. Although we think that this test might be more sensitive than the measures of awareness used in previous studies, this test does not inform us about how sure the participants are about their selection neither how pronounced they think the differences between different ranking positions are. In Experiment 3, we introduced an alternative test aimed at addressing this limitation. Specifically, we asked participants to estimate the approximate number of trials in which the singleton distractor had appeared in each location of the display (see Giménez-Fernández et al., 2020, Experiment 2).

### Method

#### Participants, apparatus, procedure, and design

Unless noted otherwise, the procedure, design, and sample size planning were identical to Experiments 1 and 2. Eighty participants took part in Experiment 3. The selection criteria and resources used to carry out the study were the same as in Experiments 1 and 2. The visual search task and the first question of the awareness test were also identical to Experiments 1 and 2. For the second question, participants were asked to estimate the number of trials in which the singleton distractor had been presented in each location of the display. The test began with the left position location (i.e., 9 o’clock), and proceeded with the remaining locations clockwise. The first location was marked with a distinctive color on the display and participants were asked “How many times do you think that the figure with a different color appeared in this particular location?” Then, this process was repeated for all the locations of the display. To avoid possible outliers, the range of possible responses was restricted to the 0–240 interval. During the test, they were able to see the responses they had already given to the previous locations. At the end of the test, participants were given the chance to repeat the test if they wanted to change their responses.

### Results and discussion

#### Attentional capture

All participants met the selection criterion. Trials were filtered following the same procedure as in Experiments 1 and 2. Figure 2 shows the average RT and accuracy in each experimental condition. As in previous experiments, the three experimental conditions differed in terms of RT, *F*(2, 158) = 341.25, *p* < .001, ${\upeta}_{\mathrm{p}}^2$ = .81, and accuracy, *F*(2, 158) = 41.83, *p* < .001, ${\upeta}_{\mathrm{p}}^2$ = .35. Participants were 86 ms faster, *t*(79) = 16.89, *p* < .001, *d*_z_ = 1.88, and more accurate, *t*(79) = 4.03, *p* < .011, *d*_z_ = 0.45, in the no-distractor than in the HPDL condition. They were also 87 ms faster, *t*(79) = 11.97, *p* < .001, *d*_z_ = 1.33, and more accurate, *t*(79) = 5.45, *p* < .001, *d*_z_ = 0.60, in the HPDL condition than in the LPDL condition. As expected, they were also significantly faster, *t*(79) = 23.83, *p* < .001, *d*_z_ = 2.66, and more accurate, *t*(79) = 8.05, *p* < .001, *d*_z_ = 0.90, in the no-distractor condition than in the LPDL condition.

#### Target suppression at the HPDL

As in Experiments 2 and 3, a non-registered analysis of RTs in no-distractor trials showed that RTs were 59 ms slower when the target was presented in the HPDL than in the remaining locations, *t*(79) = 5.55, *p* < .001, *d*_z_ = 0.62. Response accuracy, in contrast, did not differ in either types of trials, *t*(79) = 0.36, *p* = .717, *d*_z_ = 0.04.

#### Spatial gradient of the suppression effect

As shown in Fig. 3 (panel A), in distractor-present trials, RTs were significantly different depending on the distance between the current location of the distractor and the HPDL, *F*(4, 316) = 30.90, *p* < .001, ${\upeta}_{\mathrm{p}}^2$ = .28. A linear mixed-effects model with a random intercept for participants confirmed that there was a significant increase in RTs as the distance went from 0 to 4, *b* = 26.64, *t*(319) = 10.07, *p* < .001. In contrast, accuracies decreased with distance (Fig. 3, panel B), as shown by a one-way ANOVA, *F*(4, 316) = 7.98, *p* < .001, ${\eta}_p^2$ = .09, and a linear mixed-effects model, *b* = -0.003, *t*(319) = 3.05, *p* = .003.

As in Experiments 1 and 2, we also analyzed the spatial gradient of RTs (Fig. 3, panel C) and accuracies (Fig. 3, panel D) in no-distractor trials depending on the distance between the target location and the HPDL. RTs were significantly different depending on target location, *F*(4, 316) = 15.39, *p* < .001, ${\upeta}_{\mathrm{p}}^2$ = .16, and a linear mixed-effects model with random intercepts for participants confirmed that there was a decrease in RTs with distance, *b* = -16.24, *t*(319) = 6.84, *p* < .001. For accuracy rates, neither the one-way ANOVA, *F*(4, 316) = 1.44, *p* = .221, ${\upeta}_{\mathrm{p}}^2$ = .02, or the linear mixed-effects model, *b* = 0.001, *t*(319) = 1.79, *p* = .075, reached statistical significance. But note that, as in Experiments 1 and 2, performance was close to perfect in these trials.

#### Awareness test

**Fig. 7:**
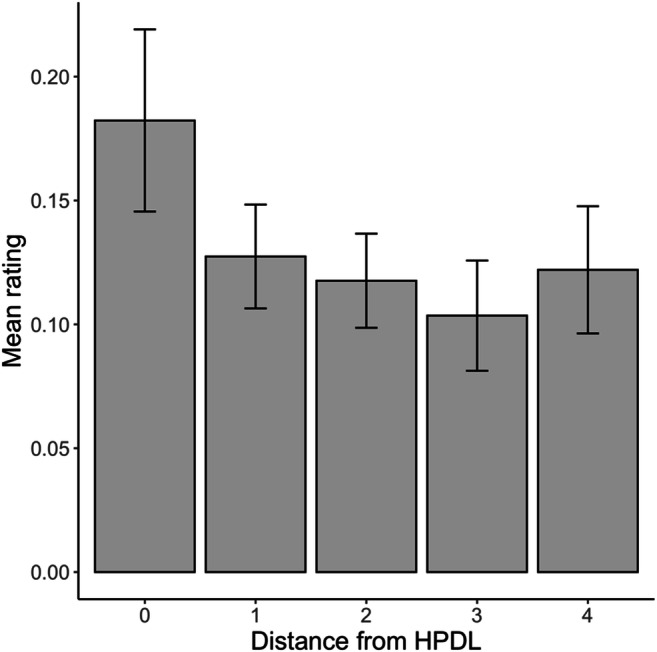
Participants’ average responses to the second question of the awareness test in Experiment 3

The responses provided by participants in response to the first question of the awareness test are shown in Fig. 4. As in Experiments 1 and 2, when asked whether they thought that the singleton tended to appear in a particular location, the modal response was “Possibly yes” (4 on a scale from 1 to 6). The average response was 3.41, 95% CI [3.20, 3.63]. Following the preregistered protocol, to analyze responses to the second question of the awareness test, we converted participants’ ratings to proportions by dividing the estimate given to each location by the sum of estimates for all locations. As in Experiment 2, we predicted that there would be a relationship between the proportion obtained by a location and its distance from the HPDL. The average proportions are shown in Fig. 7. A linear mixed-effects model with a random intercept for participants confirmed that the scores/proportion received by each location decreased significantly as the distance from the HPDL increased from 0 to 4, *b* = -0.014, *t*(637) = 5.49, *p* < .001. As in Experiments 1 and 2, these results show that participants’ ratings in the awareness test were far from random and, overall, revealed some knowledge of the spatial distribution of the singleton distractor. It is important to note, though, that the distribution of participants’ ratings did not perfectly mirror the proportion of trials in which the singleton distractor appeared in each location. Participants answered, in average, that the proportions for the HPDL were roughly between .15 and .20, while the singleton distractor appeared in the HPDL on 65% of distractor-present trials.

Interestingly, the value for location 4 is noticeably higher than for locations 2 and 3, a pattern that can be found not only in the present experiment (Fig. 7), but also in the previous one (Fig. 6). We suspect that this difference may be due to the number of data points that contribute to the calculation of each value. While the values for locations 1–3 are based on participants’ responses to two positions (e.g., the average for location 2 comprises ratings for locations +2 and -2), only one response per participant contributes to the estimate of location 4. Thus, this results in noisier, and therefore less reliable, estimates for location 4.

## Non-registered analyses

### Combined analysis of awareness tests in Experiments 1–3

**Fig. 8:**
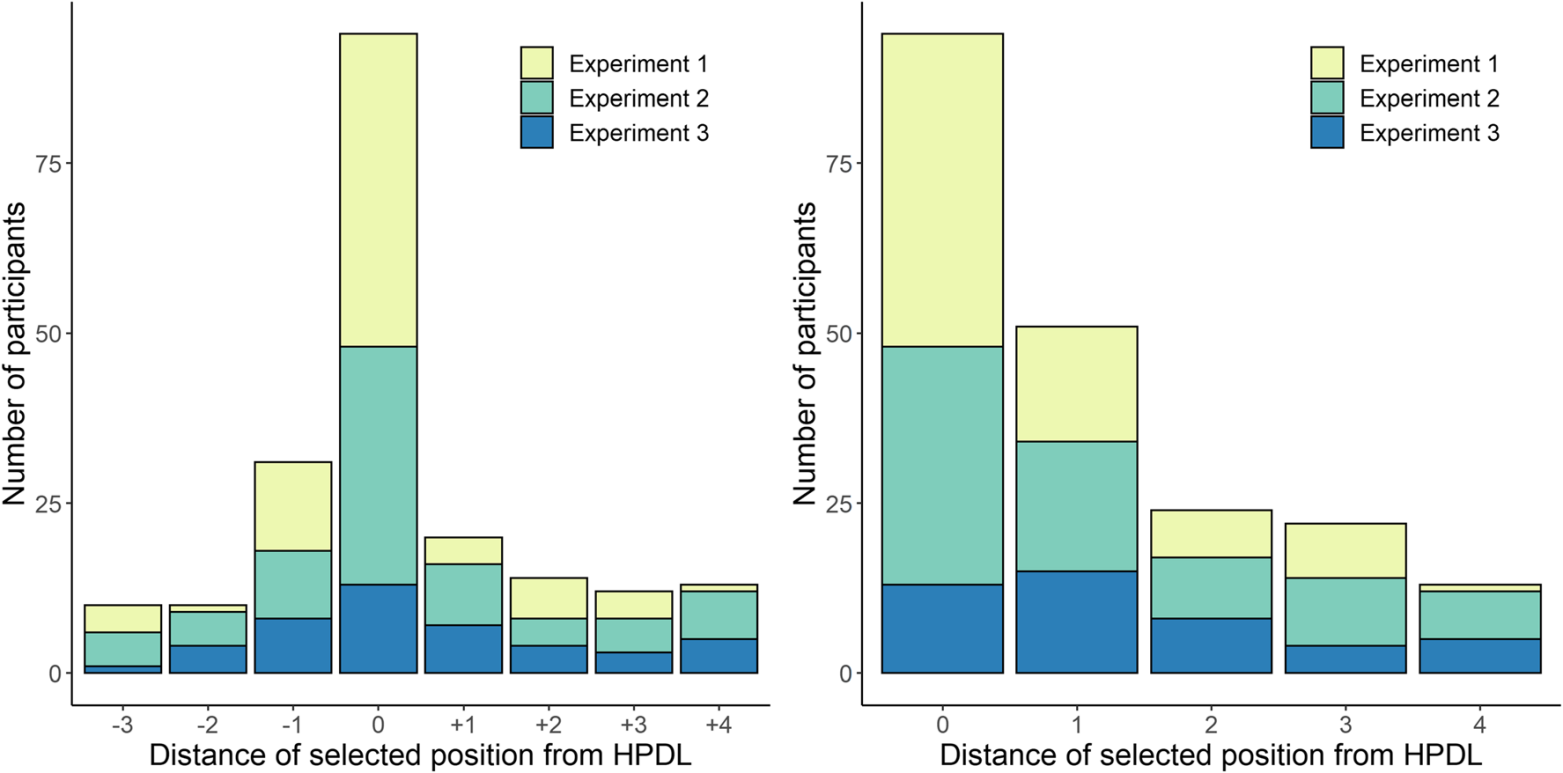
Combined analysis of participants’ responses to the second question of the awareness test in Experiments 1–3. The left panel shows the distribution of participants’ choices, where 0 represents the high-probability distractor location, and values 1–4 represent progressively distant locations. Positive and negative values indicate clockwise or counter-clockwise positions. The right panel shows participants’ choices ignoring whether they were on the clockwise or the counter-clockwise side of the high-probability distractor location (HPDL)

The awareness tests included in Experiments 2 and 3 lend themselves quite easily to the kind of analysis conducted for Experiment 1. In Experiment 2, participants were asked to select and rank the three locations that, in their opinion, had contained the singleton distractor most frequently. One would expect that if these participants had been asked to select just one location, they would have selected the location they ranked first. Similarly, in Experiment 3, participants were asked to rate the number of trials in which the singleton distractor appeared in each location. Again, one would expect that if these participants had been asked to select the location that had contained the singleton most often, they would have chosen the location for which they gave the highest rating. Following this logic, we repeated the analysis of awareness test run in Experiment 1, but this time collating data from all three experiments. For this analysis, we had to exclude data from 35 participants in Experiment 3 who gave the same (highest) rating for more than one location. The distribution of participants’ expected choices is shown in Fig. 8. The distribution of choices across locations -3, -2, -1, 0, +1, +2, +3, and +4, departed significantly from chance, *χ*^2^(7) = 223.69, *p* < .001. The distribution still differed from chance even ignoring participants who selected the HPDL, *χ*^2^(6) = 21.72, *p* = .001. As in Experiment 1, we run an additional analysis ignoring whether the selected location was on the clockwise or counter-clockwise side of the HPDL, focusing just on participants selecting locations at distance 1, 2, and 3. This test was again significant, *χ*^2^(2) = 16.23, *p* < .001. Overall, these analyses confirm that (a) participants tended to select the HPDL well above chance and (b) even when they did not, they still tended to select locations close to it.

### Analysis of participants who failed the awareness test in Experiments 2 and 3

**Fig. 9:**
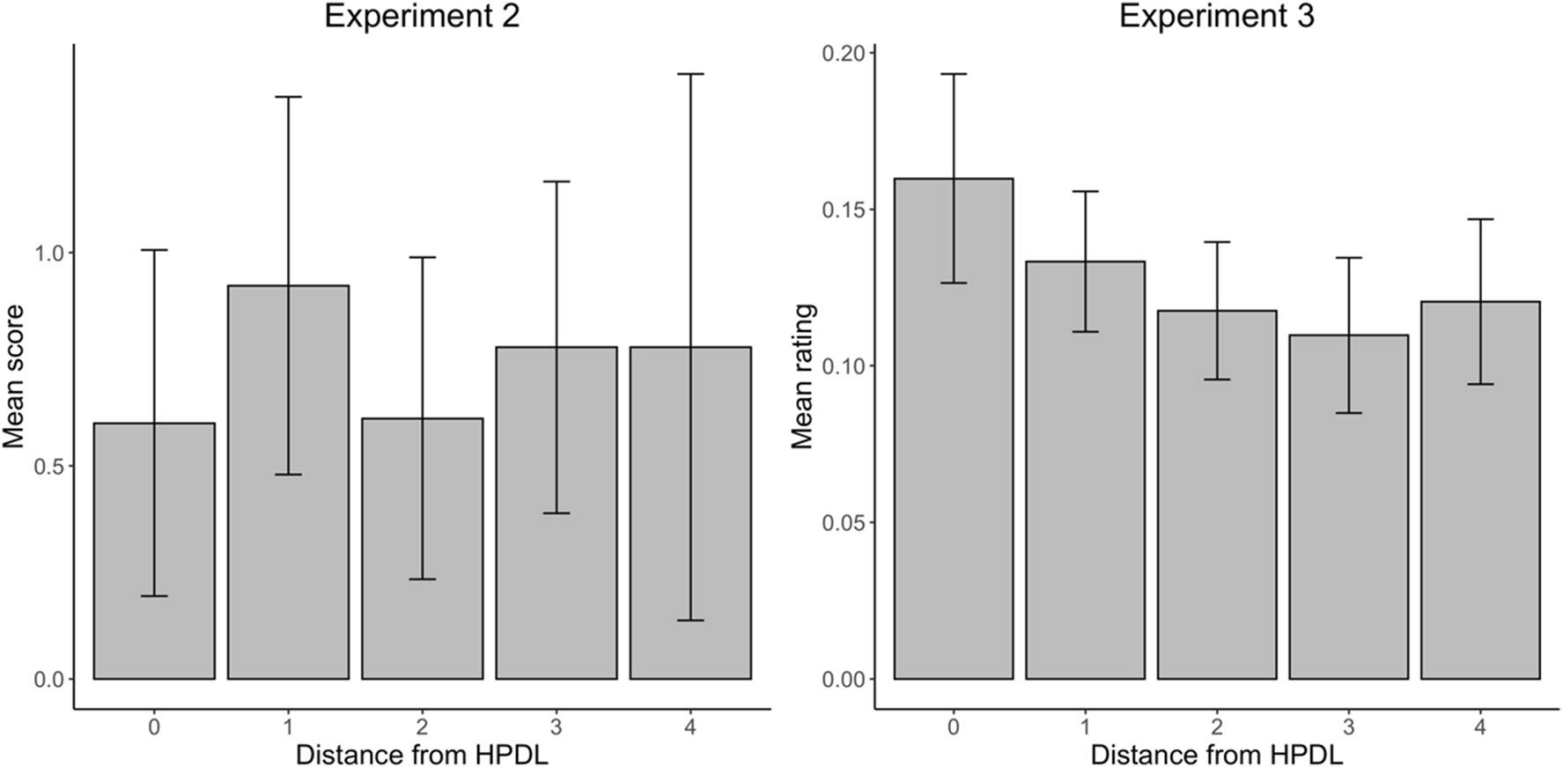
Distribution of responses to the second question of the awareness test in Experiments 2 and 3, including only participants who failed the awareness test

The preregistered protocol of Experiment 1 included separate analyses of responses in the awareness test for participants who failed to identify the HPDL. Although we did not preregister an equivalent analysis for Experiments 2 and 3, it is possible to extend the logic of those analyses to these experiments as well. In Experiment 2, we considered that participants “failed” the awareness test if they did not select the HPDL as their first option in the ranking of locations that had contained the singleton distractor most often. For Experiment 3 we considered that participants had failed the test if they did not give their highest rating to the HPDL. This included not only participants who gave their highest rating to another location, but also participants who gave the (same) highest rating to several locations, including or not the HPDL. Forty-five participants from Experiment 2 and 67 from Experiment 3 failed the awareness test according to these criteria. Their performance is summarized in the left and right panels of Fig. 9, respectively.

As can be seen, the scores obtained by each location in Experiment 2 no longer suggest a bias in favor of the HPDL among participants who failed the test. There is a slight numeric advantage for the two positions immediately next to the HPDL, but this trend is far from significance. A linear mixed-effects model, with random intercepts for participants, failed to find any significant association between the scores obtained by each location and their distance from the HPDL, *t*(358) = 0.12, *p* = .906. In Experiment 3, in contrast, there is a clear preference for the HPDL and the locations immediately next to it. A linear mixed-effects model with random intercepts for participants showed that the ratings decrease with the distance from the HPDL, *b* = 0.01, *t*(533) = 3.92, *p* < .001.

It is interesting that the decreasing gradient of ratings observed in Experiment 3 was not replicated in the ranking test of Experiment 2. Given that the learning stage was identical across the three experiments, it is unlikely that this discrepancy is due to genuine differences in participants’ awareness. Instead, these differences are more likely to depend on details of the measuring method. In our opinion, the procedure used in Experiment 3 seems to be more sensitive to detect awareness in participants who “fail” the test. A participant who fails to assign the highest rating to the HPDL in Experiment 3 can still assign a relatively large rating to this location, perhaps a value only marginally lower than their highest rating. In contrast, a participant who fails to rank the HPDL first in Experiment 2 can, at most, rank it second or third. In other words, the awareness test of Experiment 3 allows participants to provide relatively similar responses to several locations. Experiment 2, in contrast, forces participants to provide quite different responses to locations that, in their mind, might be just as likely to be the HPDL. In any case, taken together, the results of Experiments 1 and 3 suggest that even participants who do not perform perfectly in the awareness test have some explicit knowledge of the distribution of the singleton distractor.

## General discussion

In the present study, we tested the hypothesis that distractor suppression in the additional singleton task takes place outside participants’ awareness using three alternative measures of awareness in three high-powered experiments. Consistent with previous studies with the additional singleton task (Wang & Theeuwes, 2018a, 2018b), all the experiments revealed a robust distractor suppression effect. Eventually, participants learned to suppress the region of the display that was more likely to contain the singleton distractor, showing faster search times when the singleton distractor appeared in the HPDL than in the remaining locations. Contrary to the assumption that this learning takes place implicitly, we found clear evidence of awareness in all experiments. In Experiment 1, the proportion of participants who identified the HPDL was well above chance. Even those who failed to do so, tended to select locations immediately close to the HPDL. Similarly, in Experiment 2, when participants were asked to choose the three locations they thought contained the distractor more often, they chose the HPDL more often than any other location. Finally, in Experiment 3, when participants were asked to estimate how often they thought the singleton distractor had appeared in each location, their ratings were higher for the HPDL than for any other location in the display.

Some studies have claimed that this type of learning is unconscious because distractor suppression remains significant after removing data from participants who are able to identify the HPDL or because the size of the distractor suppression effect is similar for participants who can identify the HPDL and for those who can’t (Gao & Theeuwes, 2020; Wang & Theeuwes, 2018a, 2018b). However, these strategies are problematic. As mentioned above, even those participants who fail to select the HPDL in the second question of the awareness test seem to know explicitly that one region of the screen contained the distractor more often the that others. This pattern is not only found in Experiment 1, which relied on the traditional location-guessing test (see Fig. 5), but also in Experiments 2 and 3 (Figs. 8 and 9), with alternative measures of awareness. Intuitively, one would expect that if participants learn to suppress the salient distractor at the HPDL and this learning is based on conscious knowledge, then they should be able to identify the HPDL. But it is possible that over the course of the experiment participants develop alternative hypotheses about the location of the distractor that allow them to perform the task efficiently without necessarily learning that the distractors tend to appear in the HPDL. For instance, a participant might learn that the distractor tends to appear in the upper half of the screen. This hypothesis might not be sufficiently precise to identify the HPDL in the awareness test, but if the HPDL is indeed somewhere in the upper region of the screen, this participant will show some ability to suppress the salient distractor. In general, participants might have “correlated hypotheses” that may not match perfectly the experimental manipulation but still allow them to perform successfully in the task (Dulany, 1961; Vadillo et al., 2020). The fact that they lack perfect awareness of the experimental manipulation does not mean that their performance is based on unconscious learning. And, conversely, it cannot be concluded that participants who succeed at identifying the HPDL have perfect awareness of the experimental manipulation either. Surely, many of them could hit the correct response by chance and even those who have some awareness, might have only a very general and vague impression about the region where the distractor has appeared more often. When these considerations are taken into account, the argument that learning is unconscious because “unaware” participants show a robust distractor suppression effect and because their performance does not differ meaningfully from participants classified as “aware” losses much of its force.

The exclusion of “aware” participants from the analysis is often based not just on whether they can identify the HPDL in the second question of the awareness test, but also on whether they say that they did not detect any regularity in response to the first question of the awareness test (Gao & Theeuwes, 2020; Wang & Theeuwes, 2018a, 2018b). The results of our experiments (see Fig. 4) show that roughly half of the participants stated that they did not detect any regularity in the distribution of distractors. We asked participants to respond to this question using confidence ratings. Overall, participants showed very little confidence in their responses, in either direction. In principle, this could support the claim that learning was unconscious. But it is also possible that participants are just adopting a conservative criterion to respond to this question. This is a general problem of subjective measures of awareness: little confidence in the response can be due either to the lack of explicit knowledge or to a conservative bias in responding (see Dienes, 2015; Fleming & Lau, 2014; Phillips, in press; Schmidt, 2014). Although we are not aware of any systematic exploration of this problem in the particular case of the additional singleton task, we think that this interpretation is consistent with participants’ performance in the few previous studies that, to our knowledge, have collected confidence ratings. For instance, Gao and Theeuwes (2022) manipulated participants’ explicit knowledge about the statistical regularities in the additional singleton task by asking them to track the location of the singleton distractor over trials. As could be expected, the results showed a very high percentage of correct answers in the awareness test. However, and quite interestingly, when the authors measured participants’ confidence on a scale from 1 to 5, they obtained a mean response of 3.62. This suggests that even when participants have strong explicit knowledge about statistical regularities, they are still reluctant to report strong confidence in their responses, supporting the conclusion that they probably adopt a conservative criterion when responding to confidence ratings. Overall, this suggests that we should be extremely cautious when interpreting the results of these tests: neither a high score unequivocally indicates awareness, nor a low score indicates the opposite.

**Fig. 10:**
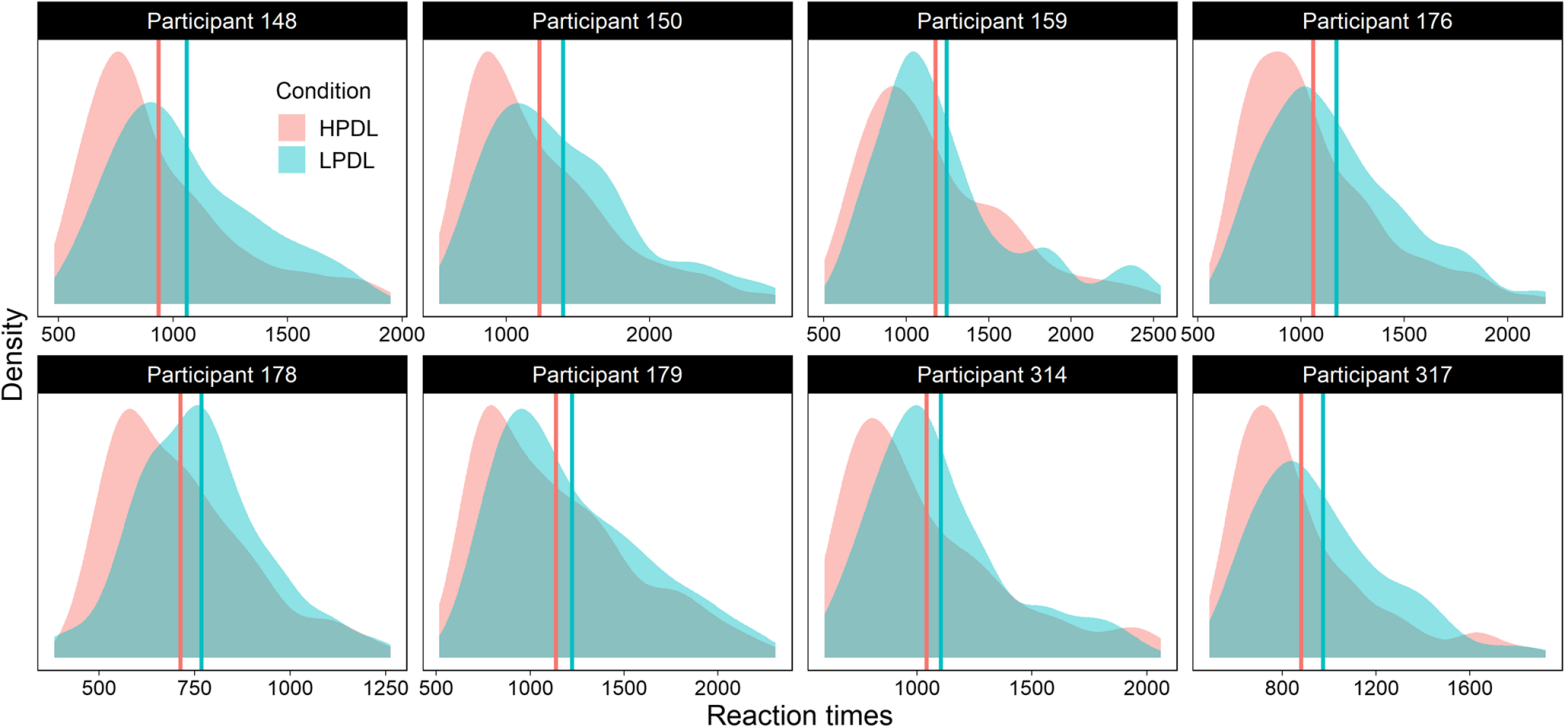
Distribution of reaction times in conditions high-probability distractor location (HPDL) and low-probability distractor location (LPDL) for eight random participants. The vertical lines denote the average reaction time in each condition. The first digit in the participants’ identification number refers to the experiment (1–3) from which they were sampled

Although we found clear evidence of awareness in our participants, it could be argued that this evidence was too weak to account for the robust distractor suppression effect observed in search times. Overall, participants were not very confident, sometimes named locations far from the HPDL, or even on the wrong side of the display, and the frequency ratings collected in Experiment 3 reveal that they greatly underestimated how often the distractor appeared in the HPDL. Doesn’t the fact that awareness is so weak prove on its own that learning must have been unconscious? In other words, if learning was entirely due to conscious processes, wouldn’t we expect clearer evidence of awareness for such a strong behavioral effect? This is an appealing argument, but it is based on a fallacy (Meyen et al., 2022; Zerweck et al., 2021). Compared to the weak effects found in the awareness test, the suppression effect detected in reaction times seems to be large and robust, but this is a misleading impression induced by the fact that suppression is measured over many trials. When explored at the trial level, the suppression effect is actually weak and unreliable. To illustrate this point, Fig. 10 shows the distribution of RTs in the two crucial conditions (HPDL vs. LPDL) for eight participants randomly sampled from Experiments 1–3. On average, all eight participants are faster in HPDL trials, but as can be seen, this advantage is quite small and accounts for little variance in the distribution of RTs. The distributions for both conditions overlap extensively, and simply knowing that a participant was relatively fast (or slow) in one trial provides very little information about whether that trial belonged to the HPDL or the LPDL condition. The small size of distractor suppression at the trial level should be taken into account when interpreting the (also weak) levels of awareness detected in the final test.

Of course, one might think that learning in the additional singleton task is driven by purely unconscious mental processes and that participants’ performance in the awareness test is above chance simply because they have some gut feeling or intuition of what the correct response might be. The fact that participants can identify the HPDL doesn’t necessarily mean that they can justify their choice or that they have any metacognitive confidence in it (e.g., Liesefeld & Muller, 2021, Appendix 5). It doesn’t mean that they used this knowledge strategically during the task either. In other words, maybe participants learn unconsciously that the singleton distractor tends to appear more frequently in one location and this (purely implicit) knowledge improves performance both during the visual search task and during the awareness test. Nothing in our data contradicts this interpretation. There is a caveat with this logic though: In most studies conducted with this task, the conclusion that learning was unconscious is based exclusively on the results of the awareness test. In our opinion, it is problematic to conclude that learning was unconscious when these tests show no evidence of awareness and yet reach the same conclusion when they show the opposite. If performance in an awareness test can be driven by either explicit or implicit knowledge, then we are forced to admit that the test provides little information about the unconscious character of learning. Ultimately, this logic leads to the conclusion that the type of awareness tests used extensively in this area should be replaced by alternative measures that are only sensitive to explicit knowledge. Otherwise, the hypothesis that performance in the task was driven by implicit processes can never be rejected.

As mentioned in the Introduction, only a handful of studies have supported the conclusion that learning in this task is unconscious using arguments independent from the results of the awareness test. For example, in the study by Wang and Theeuwes (2018a), the HPDL was cued in all trials for some participants, but this did not help them to suppress attention to the HPDL. Similarly, Gao and Theeuwes (2022) instructed some participants to track the location of the singleton distractor over the experiment. This improved performance in the awareness test, but had no noticeable effect on participants’ ability to ignore the distractor. These two experiments provide support for the hypothesis that learning in the additional singleton task is unconscious. In general, we suspect that manipulating awareness experimentally, as done in these two studies, is more likely to yield compelling evidence than simply testing whether participants perform at chance in a brief awareness test. We hope that future research will rely on this alternative strategy more often.

In summary, the results of this series of experiments do not support the conclusion that distractor suppression in the additional singleton task is driven by unconscious learning. All the experiments showed clear evidence of awareness of the statistical regularities in the spatial distribution of the singleton distractor. It has been claimed that this type of learning is unconscious because it remains significant after removing participants who fail the test of awareness. However, our results suggest that this strategy is problematic. Participants who fail to identify the HPDL in the test of awareness are still very likely to select a location close to it, suggesting that they nevertheless have some explicit knowledge of the biased distribution of distractors. Moreover, although many participants state that they have not detected any regularity in the task, they show very little confidence in their responses. Overall, it is unclear whether these subjective judgments are genuinely due to unconscious learning or simply to a conservative response criterion. Future research should address this question using alternative measures of awareness and analytic approaches that can disentangle response biases from genuine awareness.

## Supplementary information

ESM 1 (DOCX 32 kb)

## Footnotes

1 Note that in the literature it is not uncommon to use the terms “goal-directed”, “top-down”, and “voluntary” as near synonyms (Theeuwes, 2018). In this article, we follow Gaspeling and Luck (2018) and use the term “goal-directed” to refer to the kind of processes denoted as “top-down” by Theuwees (2018).

2 As can be seen in Fig. 3, the relationship between RTs (or accuracies) and distance from the HPDL does not seem to follow a perfectly linear function. However, we analyzed the data with a linear function following the preregistered protocol, which, in turn, was based on the analyses reported by Wang and Theeuwes (2018b).
