# Supplementary material for: Learning to suppress a distractor may not be unconscious
Source: Atten Percept Psychophys. 2022 Nov 23;85(3):796–813. doi: 10.3758/s13414-022-02608-x (PMC10066157; doi:10.3758/s13414-022-02608-x)
Supplement: Supplementary file 1 — (DOCX 32 kb) [file 13414_2022_2608_MOESM1_ESM.docx]

**SUPPLEMENTARY MATERIAL**

**Learning to suppress a distractor may not be unconscious**

Francisco Vicente-Conesa, Tamara Giménez-Fernández,

David Luque, & Miguel A. Vadillo

**Awareness measurement in the previous literature**

In the main text we argue that most studies conducted with the additional singleton task measure awareness including only two questions, one addressing whether participants noticed a bias in the distribution of distractors and a second one testing whether they are able to identify the HPDL, even if they have to guess. To confirm that this is actually the case in most studies, we conducted a brief literature search to summarize the different ways in which awareness had been measured so far in this literature. It is important to note that this does not intend to be a systematic review about this topic. Specifically, to simplify the literature search, we selected all the papers citing Wang and Theeuwes (2018) in Google Scholar that met the following selection criteria: a) used the additional singleton task, b) manipulated the probability of the singleton distractor or the target appearing in different locations, and c) measured participants’ awareness about those regularities. A total of 26 papers matched the mentioned criteria. Table S1 summarizes the procedure of the awareness tests used in these studies, quoting the relevant information from the Method or Results sections of each paper.

As can be seen, the majority of studies do follow the two-question structure outlined above: First they test whether the participants were able to identify any regularity in the presentation of the stimuli and the they collect more specific information about that regularity (usually participants are asked to indicate which location had contained the distractor most often). Only a few studies ask participants to rate the confidence in their answers. Furthermore, sample sizes are often too small to reach high power levels.

| **Table S1** | | |
| --- | --- | --- |
| *Summary of the awareness tests used in the literature* | | |
| **Studies** | **Procedure for awareness test** | **Sample size** |
| Wang & Theeuwes (2018) | “Following the experiment, participants were asked to indicate the location that they thought contained the distractor most often and how confident they were of their answers (scaled from 1 to 7).” p. 16 | *N* = 32 |
| Allenmark et al. (2019) | “This involved a three-stage procedure: first, participants had to indicate whether the distractor distribution was centred on one of four specific regions: top, bottom, left and right, or equal across the four different regions; second, (even when they had given an “equal” response in stage 1), participants had to give a forced-choice response: indicating in which of the four regions the distractor had occurred most frequently (by marking the corresponding region on the “display” depicted on the answer sheet); Finally, participants were to report how confident they were in the above answers using a seven-point rating scale, with a rating of 1 representing “Not at all confident” and 7 “Extremely confident.”” p. 581 | *N* = 30 + 30 + 30 + 30 |
| Di Caro et al. (2019) | “At the end of the experimental session we formally assessed through a brief questionnaire whether participants had become aware of the biased probability of distractor location. They were first asked to report whether they thought they had noticed something peculiar about the spatial distribution of the distractor and, second, to report/guess the location/s where they thought it appeared most frequently.” p. 147 | *N* = 30 |
| Failing et al. (2019a) | “After performing the task, participants were required to answer three forced-choice questions as part of a final implicit learning questionnaire […]. For the first question, they were informed that the distractors displayed certain regularities and were asked to indicate whether they had noticed a regularity. For the final two questions, they were explicitly told that one particularly colored distractor was more likely to appear in one, while the other particularly colored distractor was more likely to appear in another location of the search display. Following this information, they were asked to indicate the locations for both distractors separately.” p. 4 | *N*_Exp1_ = 48  *N*_Exp2_ = 48 |
| Failing et al. (2019b) | “After performing the search task, participants were asked to answer two questions. For the first question, they were required to indicate whether they were aware that the target was presented less often in one particular location. If they answered with “yes” they had to indicate that location on an illustration of the search display. For the final question, they were asked to indicate the confidence about their answer on a 6-point scale (going from not confident at all, i.e., 0% sure, to very confident, i.e., 100% sure).” p. 1407 | *N*_Exp1_ = 24  *N*_Exp2_ = 24 |
| Van Morselaar et al. (2019) | “At the end of the experiment participants were asked whether they noticed that one location contained a distractor with higher probability than other locations, and if so to indicate which location was the high probability location.” p. 345 | *N*_Exp1_ = 18  *N*_Exp2_ = 66 |
| Wang et al. (2019) | “To examine if observers noticed the statistical regularities, we asked observers to complete a questionnaire after the experiment was concluded that contained a depiction of eight circles placed on the radius of an imaginary circle. Observers had to report whether they thought the color singleton distractor was presented more often at a specific location and, if so, to mark that location. Furthermore, they were asked to indicate their level of certainty regarding their decision on a scale of 1–7 (7 being the highest).” p. 1818 | *N* = 16 |
| Zhang et al. (2019) | “This involved a two-stage procedure: first, participants had to indicate whether the distractor distribution was equal across all locations, or centered on one specific location; second (even when they had given an equal response in stage 1) participants had to give a forced-choice response at which of the 8 locations the distractor had occurred most frequently (by marking the corresponding location on the display depicted on the answer sheet).” p. 1150 | *N*_Exp1_ = 24  *N*_Exp2_ = 24  *N*_Exp3_ = 24 |
| Failing & Theeuwes (2020) | “After the experiment, each participant had to fill in an implicit learning questionnaire querying them with three questions about the distractors and their spatial regularities. For the first question, participants were informed that the distractors displayed certain regularities and were asked to indicate which, if any, they had noticed. For the last two questions, they were explicitly told that the high-salience distractor was more likely to appear in one while the low-salience distractor was more likely to appear in another location, and then asked to indicate these locations for each distractor separately.” p. 88 and 89 | *N*_Exp1_ = 24  *N*_Exp2_ = 24 |
| Gao & Theeuwes (2020) | “[P]articipants were asked to answer two questions after the whole experiment. They were asked to indicate if they had noticed any regularity regarding the location of the distractor, and irrespective of their answer had to indicate one location in each session on the search display where they thought that the distractor appeared more often.” p. 99 | *N*_Exp1_ = 24  *N*_Exp2_ = 24 |
| Li & Theeuwes (2020) | “After finishing the whole task, 22 participants were required to recall whether they were aware of any regularities regarding target locations between trials and write down what kind of regularity they were aware of. The other 12 participants were asked to complete an eight-alternative forced-choice task to choose at which location the target was most likely to appear after the first item of the regularity pairs. Specifically, we showed participants a search array consisting of a diamond (target at the predicting location) among seven circles on the left side of the display and an array of eight circles representing the eight locations on the right side of the display. Participants were asked to choose the location in the array on the right side that they thought the target was most likely to appear following the search array that was displayed on the left side of the display. The same question was asked for the other regularity pair.” p. 3 and 4 | *N*_Exp1_ = 22 + 12  *N*_Exp2_ = 34  *N*_Exp3_ = 22 + 13  *N*_Exp4_ = 23 + 11 |
| Wang & Theeuwes (2020) | “Following the main experiment, participants had to indicate those locations that they thought contained the distractor singleton most often during the experiment by answering the question, “Did you notice that the distractor singleton always appeared at one or multiple locations? If yes, which location (s)?”. They were also asked to indicate their confidence about their answer (scaled from 1 to 7; 1 means 100% sure and 7 means 100% unsure) by pressing the corresponding buttons on the keyboard.” p. 3 of the Supplementary Material | *N*_Exp1_ = 24  *N*_Exp2_ = 24 |
| Gong & Theeuwes (2021) | “After the experiment, participants were required to fill in an implicit learning questionnaire with two forced-choice questions. For the first question, they were asked whether they noticed any regularities regarding the locations where the distractors were presented. For the second question, they were explicitly informed that there was one location that had a larger probability to display a distractor, and were asked to indicate that location.” p. 296 | *N*_Exp1_ = 31  *N*_Exp2_ = 24  *N*_Exp3_ = 38 |
| Huang et al. (2021) | “After the experiment, participants’ awareness regarding the statistical regularities of the distractor location was assessed. They were asked if they were aware of the high-probability location of the distractor, and if so, they were asked to mark which location that was and to express their confidence in the answer on a seven-point Likert scale (1 = very doubtful, 7 = very confident).” p. 6 | *N* = 60 |
| Lin et al. (2021) | “After performing the search task, we tested participants’ awareness regarding the high-probability location. They had to answer three questions: (1) They needed to indicate whether they were aware that the distractor was presented more often in one particular location. (2) If they answered “yes,” they had to indicate which location was the high-probability location; if they answered “no,” they had to guess the high-probability location. (3) They were asked to indicate the confidence in their answer on a 7-point scale (from not confident, i.e., 0% sure, to very confident, i.e., 100% sure).” p. 286 | *N* = 16 + 16 + 16 + 16 + 16 + 16 +16 |
| Van Morselaar et al. (2021) | “At the end of the second session, a display with only circles, each with a unique number (i.e., 1-8) corresponding to one of the search locations was shown. Participants were asked to indicate (and if necessary, guess) which location they believed had contained the singleton distractor most frequently throughout the experiment.” p. 235 | *N* = 24 |
| Van Morselaar & Theeuwes (2021) | “After the last block, participants were first asked whether they noticed that one location had a higher distractor probability. Subsequently, a display with white circles, each with a unique identifier, corresponding to one of the search locations was shown, and participants had to indicate (and, if necessary, guess) which location they believed contained the singleton distractor most frequently throughout the experiment.” p. 3 | *N* = 48 |
| Wang et al. (2021) | “After the experiment, participants of the learning group were told that some elements in the display were consistently placed at particular locations within the display. Participants were asked whether they noticed these regularities, and if they did, whether they could tell what they had noticed.” p. 2746 | *N*_Exp1_ = 48  *N*_Exp2_ = 48 |
| Xu et al. (2021) | “At the end of the experiment, all participants were asked to indicate at which two locations they thought the color singleton distractors appeared most often and whether it occurred after a short or after a long interval.” p. 1060 | *N* = 35 |
| de Waard et al. (2022) | “Awareness of the spatial regularities was assessed after all trials were completed by asking participants whether the distractor appeared more frequently in one location, and secondly to indicate this location in four trials (context A/B × circle/diamond-shaped target) by typing in a location-based number (1–8).” p. 462 | *N*_Exp1_ = 61  *N*_Exp2_ = 114  *N*_Exp3_ = 60 |
| Duncan et al. (2022) | “Following the completion of the experiment, the participant was asked to answer an additional four debrief questions (for three participants the debrief was collected verbally). Firstly, they were asked if they noticed the target tended to appear in certain locations more frequently than others. Secondly, they were asked to indicate where they believed the target was most frequently present on the final experiment block which they had just completed. If they were not sure they were instructed to provide a best guess. Thirdly they were asked if they felt any of the other seven locations at some point in the experiment was more likely to hold a target. Finally, the participant was asked if they had ever performed a task similar to the one they had just finished.” p. 15 | *N* = 24 |
| Gao & Theeuwes (2022) | “At the end of the experiment, participants of the two different groups were asked to answer three same questions. First, they were asked whether they noticed that there was a specific location at which distractor appeared more often (yes or no). Second, they needed to indicate the exact location by pressing the corresponding number (1–8; the display here was similar to the report display). For the last question, we asked them how confident they were about their answers (scaled from 1 to 5).” p. 1090 | *N*_Exp1_ = 40 + 40  *N*_Exp2_ = 40 + 40 |
| Huang et al. (2022) | “After the experiment, participants’ awareness regarding the statistical regularities of the target location was assessed. Participants were asked if they were aware that one location contained the target more often than any of the other locations, and indicate which location they thought contained the target more often (they had to specify this location regardless of whether they had indicated that they noticed the regularity.” p. 5 | *N*_Exp1_ = 72  *N*_Exp2_ = 72  *N*_Exp3_ = 180 |
| Kerzel et al. (2022) | “At the end of the experiment, we asked all participants to indicate a high-frequency distractor position. Participants were shown an illustration indicating the eight stimulus locations and marked the location where they thought the distractor had been presented more frequently.” p. 1893. | *N* = 40 |
| Van Morselaar & Theeuwes (2022) | “After the last block, participants were first asked whether they noticed that one location had a higher distractor probability. Subsequently, a display with white circles, each with a unique identifier, corresponding to one of the search locations was shown and participants had to indicate (and if necessary, guess) which location they believed contained the singleton distractor most frequently throughout the experiment.” p. 452 | *N* = 40 |
| Zhang et al. (2022) | “After performing the search task, we tested participants’ awareness regarding the high-probability target location in the groups with the overall probability of 40%, 50%, 70%, 80%, and 90%. They had to answer three questions: (1) They need indicate whether they were aware that the target was presented more often in one particular location. (2) If they answered with “yes,” they had to indicate which location was high probability location; if they answered with “no,” they had to guess the high-probability location. (3) They were asked to indicate the confidence about their answer on a 7-points scale (ranging from not confident at all, i.e., 0% sure, to very confident, i.e., 100% sure).” p. 1079 | *N* = 16 + 16 + 16 + 16 + 16 |
| *Note.* When a study comprises several experiments, their *N* is reported separately for each experiment using subscripts. If a experiment comprises several groups, the *N* for each group is reported separately using the “+” sign. | | |

**Intertrial Priming in Experiments 1-3**

Following Wang and Theeuwes (2018), our preregistered protocol included a series of analyses to ensure that the effects detected in the main analyses were driven by the locations of stimuli, and not by other features like their color. Specifically, we tested whether reaction times in the HPDL condition were significantly lower when the distractor appeared in the same color as in the previous trial. Because these analyses are somewhat tangential to the main goal of Experiments 1-3, we report them only in the present Supplementary Material.

In Experiment 1, a paired-samples *t-*test comparing RTs in HPDL trials with the singleton distractor presented in the same color as in the previous trial with RTs in HPDL trials with the distractor presented in a different color was not significant, *t*(78) = 0.38, *p* = .708, *d*_z_ = 0.04. To test whether this non-significant result provides genuine support for the null hypothesis, we computed the Bayes factor comparing the evidence in favor of the null hypothesis with an alternative hypothesis modelled as a Cauchy distribution with the scaling parameter set to .707. The BF_01_ was 7.54, providing moderate evidence that the repetition of the singleton color did not make a difference in RTs for HPDL trials. We performed the same analysis on the proportion of correct responses. Accuracy in HPDL trials did not differ depending on whether the color of the singleton distractor repeated or not, *t*(78) = 0.77, *p* = .446, *d*_z_ = 0.08, and the Bayes factor analysis also returned moderate evidence in favor of the null hypothesis, BF_01_ = 6.08. These results suggest that performance in HPDL trials did not benefit from repeating the color of the singleton distractor, lending support for the hypothesis that these effects are driven by the location of the singleton distractor.

In contrast, in Experiment 2, RTs in the HPDL condition were significantly faster when the singleton distractor was presented in the same color as in the previous trial, *t*(79) = 2.12, *p* = .037, *d*_z_ = 0.23. The Bayes factor analysis showed that this result did not provide clear support for the alternative over the null hypothesis, BF_10_ = 1.02. The same effect was found for accuracy rates, *t*(79) = 2.72, *p* = .008, *d*_z_ = 0.30, although in this case the Bayesian analysis did provide moderate support for the alternative hypothesis, BF_10_ = 3.78.

Similarly, in Experiment 3, RTs in the HPDL condition were significantly faster when the singleton distractor was presented in the same color as in the previous trial, *t*(79) = 3.64, *p* < .001, *d*_z_ = 0.40, and the Bayes factor analysis provided strong support for the alternative hypothesis, BF_10_ = 46.60. The same effect was found for accuracy rates, *t*(79) = 2.69, *p* = .009, *d*_z_ = 0.30, providing moderate support for the alternative hypothesis, BF_10_ = 3.50.

Given that the results of Experiments 2 and 3 suggest that in HPDL trials participants were slightly faster and more accurate when the color of the singleton distractor was the same as in the previous trial, we conducted a (non-registered) combined analysis of the intertrial priming effect on RTs in Experiments 1-3, entering Experiment as an additional factor. While the main effect of color repetition was significant, *F*(1, 236) = 12.79, *p* < .001, $\eta_{p}^{2}$ = .05, the main effect of Experiment and the Repetition × Experiment interaction were far from statistical significance, *F*(2, 236) = 0.12, *p* = .887, $\eta_{p}^{2}$ = .001, and *F*(2, 236) = 2.52, *p* = .082, $\eta_{p}^{2}$ = .02, respectively. On average, RTs were 12.45 ms faster, 95% CI [5.55, 19.35], when the color of the distractor repeated. Note that this effect is much smaller than the average difference between the HPDL and LPDL conditions across Experiments 1-3, namely, 82.97 ms, 95% CI [75.97, 89.96], suggesting that the location-based suppression effect is much stronger than feature-based inter-trial priming. To confirm that location-based suppression remained significant even after controlling for inter-trial priming, we compared RTs in the LPDL and HPDL conditions in a two-way ANOVA with Repetition and Condition (LPDL and HPDL) as factors, collating data from the three experiments. This ANOVA yielded significant main effects of Repetition, *F*(1, 238) = 21.55, *p* < .001, $\eta_{p}^{2}$ = .08, and Condition, *F*(1, 238) = 499.94, *p* < .001, $\eta_{p}^{2}$ = .68. But the Repetition × Condition interaction failed to reach statistical significance, *F*(1, 238) = 0.27, *p* = .604, $\eta_{p}^{2}$ = .001. To quantify the amount of support for the null hypothesis in the Repetition × Condition interaction, we conducted a Bayesian ANOVA using the ‘BayesFactor’ R package, with the default settings. The best fitting model included the main factors of Repetition and Condition, but not the interaction. The Bayes factor of this model over the model including also the interaction was 9.41, providing moderate to strong evidence against the interaction. Therefore, although visual search benefitted from intertrial priming, this effect had roughly the same size in HPDL and LPDL trials and cannot account for the difference between them.

**Awareness Questions in the Original Language**

In this section we report the original text (in Spanish) used in the awareness test. In all the experiments, the awareness test included two questions. First, participants were asked if they noticed any statistical regularity related to the location of the distractor: “Habrás observado que, en muchos ensayos, aparecía una figura de un color diferente al resto. ¿Te ha parecido que esa figura apareció con más frecuencia en algún lugar concreto de la pantalla?”. Participants were able to select answers from 1 to 6 labelled “seguro que no”, “probablemente no”, “creo que no”, “creo que sí”, “probablemente sí” and “seguro que sí”. In Experiment 1, the second question of the test was: “A continuación, se te pedirá que elijas la localización en la que crees que la figura de diferente color ha aparecido con más frecuencia.”. In Experiment 2, the second question of the test comprised three separate trials or questions. In the first one, they were asked: “A continuación, se te pedirá que elijas la localización en la que crees que la figura de diferente color ha aparecido con más frecuencia.”. Right after this, they were asked: “Ignorando la respuesta anterior, ¿cuál es la siguiente localización en la que crees que ha aparecido con más frecuencia la figura de distinto color?”. And finally, they were asked: “Por último, ignorando las respuestas anteriores, ¿cuál es la siguiente localización que crees que ha albergado más veces la figura de distinto color?”. In Experiment 3, in the second question of the test they were asked to estimate the number of trials in which singleton distractor was presented each of the eight locations of the display: “A continuación, se te pedirá que hagas una estimación numérica (de 0 a 240) sobre la frecuencia con la que la figura diferente ha aparecido en cada una de las localizaciones.”. After this, they were able to read the next question: “Teclea el número de veces (entre 0 y 240) que crees que ha aparecido la figura con distinto color en la localización marcada y pulsa enter.”

**References**

Allenmark, F., Zhang, B., Liesefeld, H. R., Shi, Z., & Müller, H. J. (2019). Probability cueing of singleton-distractor regions in visual search: The locus of spatial distractor suppression is determined by colour swapping. *Visual Cognition, 27,* 576-594.

de Waard, J., Bogaerts, L., van Moorselaar, D., & Theeuwes, J. (2022). Surprisingly inflexible: Statistically learned suppression of distractors generalizes across contexts. *Attention, Perception, & Psychophysics, 84,* 459-473.

de Waard, J., Bogaerts, L., van Moorselaar, D., & Theeuwes, J. (2022). Surprisingly inflexible: Statistically learned suppression of distractors generalizes across contexts. *Attention, Perception, & Psychophysics, 84,* 459-473.

Di Caro, V., Theeuwes, J., & Della Libera, C. (2019). Suppression history of distractor location biases attentional and oculomotor control. *Visual Cognition, 27*, 142-157.

Duncan, D., van Moorselaar, D., & Theeuwes, J. (2022). Pinging the brain to reveal the hidden attentional priority map. *bioRxiv.*

Failing, M., & Theeuwes, J. (2020). More capture, more suppression: Distractor suppression due to statistical regularities is determined by the magnitude of attentional capture. *Psychonomic Bulletin & Review, 27*(1), 86-95.

Failing, M., Feldmann-Wüstefeld, T., Wang, B., Olivers, C., & Theeuwes, J. (2019a). Statistical regularities induce spatial as well as feature-specific suppression. *Journal of Experimental Psychology: Human Perception and Performance, 45*, 1291.

Failing, M., Wang, B., & Theeuwes, J. (2019b). Spatial suppression due to statistical regularities is driven by distractor suppression not by target activation. *Attention, Perception, & Psychophysics, 81*, 1405-1414.

Gao, Y., & Theeuwes, J. (2020). Learning to suppress a distractor is not affected by working memory load. *Psychonomic Bulletin & Review, 27*, 96-104.

Gao, Y., & Theeuwes, J. (2022). Learning to suppress a location does not depend on knowing which location. *Attention, Perception, & Psychophysics, 84*, 1087–1097.

Gong, D., & Theeuwes, J. (2021). A saliency-specific and dimension-independent mechanism of distractor suppression. *Attention, Perception, & Psychophysics, 83*, 292-307.

Huang, C., Donk, M., & Theeuwes, J. (2022). Proactive enhancement and suppression elicited by statistical regularities in visual search. *Journal of Experimental Psychology: Human Perception and Performance, 48,* 443-457.

Huang, C., Vilotijević, A., Theeuwes, J., & Donk, M. (2021). Proactive distractor suppression elicited by statistical regularities in visual search. *Psychonomic Bulletin & Review, 28*, 918-927.

Kerzel, D., Balbiani, C., Rosa, S., & Huynh Cong, S. (2022). Statistical learning in visual search reflects distractor rarity, not only attentional suppression. *Psychonomic Bulletin & Review, 29,* 1890-1897.

Li, A. S., & Theeuwes, J. (2020). Statistical regularities across trials bias attentional selection. *Journal of Experimental Psychology: Human Perception and Performance, 46*, 860.

Lin, R., Li, X., Wang, B., & Theeuwes, J. (2021). Spatial suppression due to statistical learning tracks the estimated spatial probability. *Attention, Perception, & Psychophysics, 83*, 283-291.

van Moorselaar, D., & Theeuwes, J. (2021). Statistical distractor learning modulates perceptual sensitivity. *Journal of vision, 21*, 3.

van Moorselaar, D., & Theeuwes, J. (2022). Spatial suppression due to statistical regularities in a visual detection task. *Attention, Perception, & Psychophysics, 84,* 450-458.

van Moorselaar, D., Daneshtalab, N., & Slagter, H. A. (2021). Neural mechanisms underlying distractor inhibition on the basis of feature and/or spatial expectations. *Cortex, 137,* 232-250.

van Moorselaar, D., Theeuwes, J., & Olivers, C. N. (2019). Memory-based attentional biases survive spatial suppression driven by selection history. *Visual Cognition, 27,* 343-350.

Wang, B., & Theeuwes, J. (2018b). Statistical regularities modulate attentional capture. *Journal of Experimental Psychology: Human Perception and Performance*, *44*, 13–17.

Wang, B., & Theeuwes, J. (2020). Implicit attentional biases in a changing environment. *Acta Psychologica, 206,* 103064.

Wang, B., Samara, I., & Theeuwes, J. (2019). Statistical regularities bias overt attention. *Attention, Perception, & Psychophysics, 81*, 1813-1821.

Wang, L., Wang, B., & Theeuwes, J. (2021). Across-trial spatial suppression in visual search. *Attention, Perception, & Psychophysics, 83,* 2744-2752.

Xu, Z., Los, S. A., & Theeuwes, J. (2021). Attentional suppression in time and space. *Journal of Experimental Psychology: Human Perception and Performance, 47*, 1056-1062.

Zhang, B., Allenmark, F., Liesefeld, H. R., Shi, Z., & Müller, H. J. (2019). Probability cueing of singleton-distractor locations in visual search: Priority-map- versus dimension-based inhibition? *Journal of Experimental Psychology: Human Perception and Performance, 45,* 1146-1163.

Zhang, Y., Yang, Y., Wang, B., & Theeuwes, J. (2022). Spatial enhancement due to statistical learning tracks the estimated spatial probability. *Attention, Perception, & Psychophysics, 84,* 1077-1086.
